# Force Distribution and Contact Mechanics in Mini-Tablet Compaction: A Discrete Element Method Study

**DOI:** 10.1007/s11095-026-04127-y

**Published:** 2026-06-18

**Authors:** Saeed Najafian, Trong Bien Tran, Bodhisattwa Chaudhuri, Tze Ning Hiew

**Affiliations:** 1 Department of Pharmaceutical Sciences, School of Pharmacy and Pharmaceutical Sciences, University of Connecticut, Storrs, CT 06269 USA; 2 Department of Chemical and Biomolecular Engineering, College of Engineering, University of Connecticut, Storrs, CT 06269 USA; 3 Department of Pharmaceutical Sciences and Experimental Therapeutics, College of Pharmacy, The University of Iowa, Iowa City, IA 52242 USA; 4 Institute of Material Sciences, University of Connecticut, Storrs, CT 06269 USA

**Keywords:** compaction, die filling, discrete element method (DEM), force chains, mini-tablet

## Abstract

**Purpose:**

The purpose of this study was to investigate how powder fill weight, particle size, and particle size distribution influence force transmission and compaction behavior of powders during compaction within narrow die cavities.

**Methods:**

Discrete element method simulations were used to model confined powder compaction. Monodisperse and polydisperse systems were analyzed by varying particle size, size distribution breadth, and fill weight. Compaction force–displacement behavior was examined together with particle-level normal, tangential, and cohesive forces, including their axial and radial spatial distributions.

**Results:**

Particle size had a stronger influence on force heterogeneity than fill weight within the range examined, with larger particles generating higher and more heterogeneous contact forces. Force distributions were positively skewed, indicating load localization within force chains. Normal and tangential forces were highest near the die wall, reflecting strong confinement effects. While individual particle forces followed similar size-dependent trends across different size distributions, narrow distributions required higher compaction forces due to localized load-bearing structures, whereas wider distributions promoted more uniform force sharing and lower resistance to compaction.

**Conclusions:**

The results demonstrated that macroscopic compaction behavior was governed by the organization of force networks rather than particle-scale force magnitudes alone, highlighting the critical roles of particle size, polydispersity, and confinement effects in powder compaction.

**Graphical abstract:**

**Figure:**
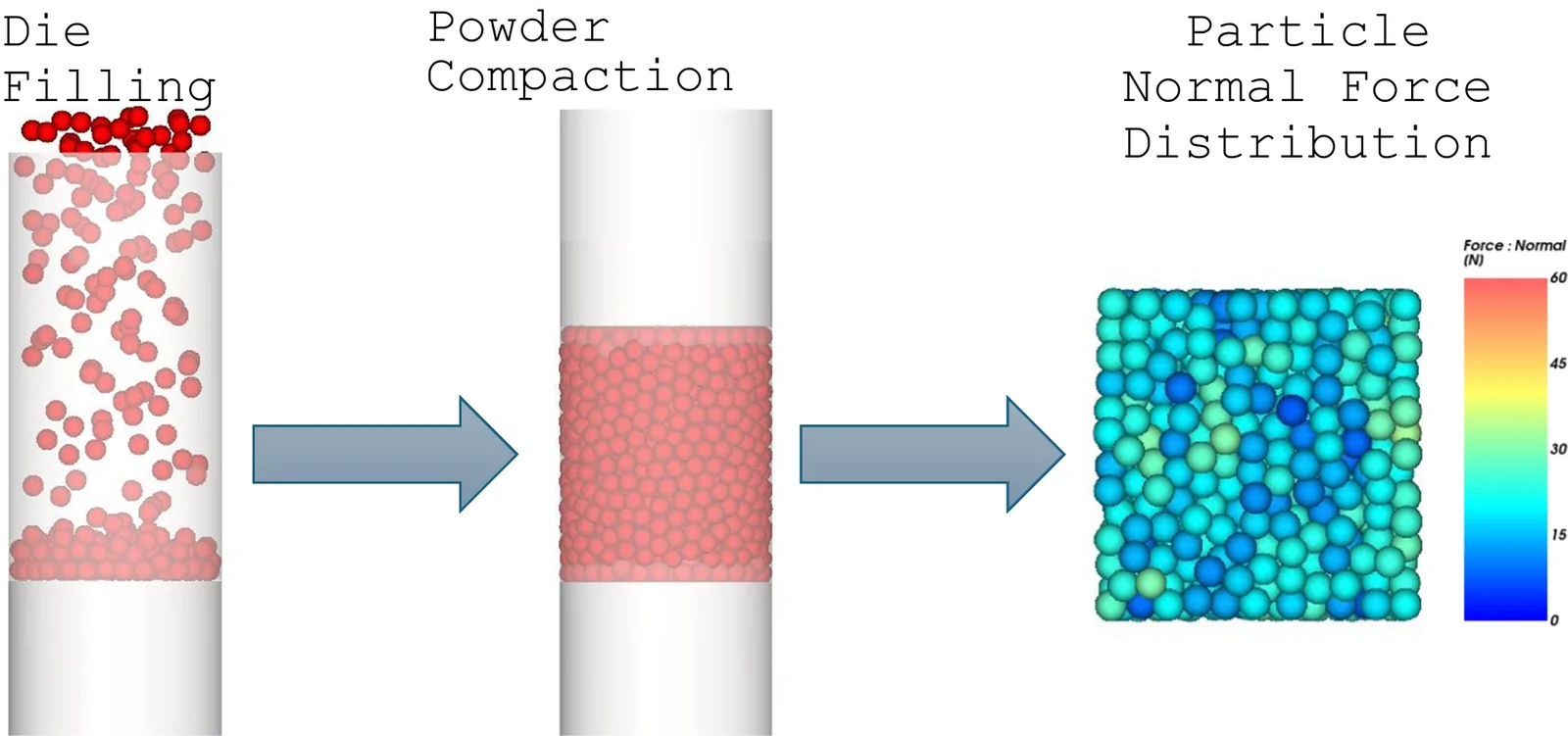

**Supplementary Information:**

The online version contains supplementary material available at https://doi.org/10.1007/s11095-026-04127-y.

## Introduction

In recent years, there has been an increased demand in the pharmaceutical industry to produce smaller compacts with diameters below 4 mm, collectively known as mini-tablets [1]. The shift towards smaller dosage forms is motivated by several factors, with a major driver being the growing emphasis on personalized medicine [2–4]. Smaller dosage units are essential in personalized medicine to enable flexible and safe treatment tailored to the unique needs of each patient [5–10]. While there are other dosage forms that are similarly small and amenable to personalized medicine (e.g., pellets), mini-tablets inherit the advantages of conventional sized tablets including good stability, low production cost, high throughput, and ease of handling and packaging [11, 12]. Mini-tablets have been shown to exhibit better compactibility than larger tablets [13, 14], enabling poorly compactible active pharmaceutical ingredients (APIs), for which the manufacture of large-sized tablets was suboptimal, to be produced as mini-tablets with acceptable mechanical strength [14]. Moreover, compared to other multi-particulate dosage forms such as pellets and granules, mini-tablets are highly reproducible in size, weight, and solid fraction [15, 16]. The smooth surfaces of mini-tablets are also favorable for subsequent film coating, leading to improved coat uniformity, reduced loss of coating materials, and lower overall production costs [17, 18]. Mini-tablets can further be developed as specialized dosage forms to meet specific therapeutics needs, including orally disintegrating mini-tablets [19, 20], bioadhesive mini-tablets [21], ocular inserts [22], modified- or sustained-release systems [16, 17, 23], and other customized drug delivery systems [2, 4]. However, due to their reduced dimensions, mini-tablet manufacturing is more complex and challenging relative to larger tablets. Consequently, the full potential of mini-tablets has not been realized because of stringent requirements related to manufacturing, characterization, clinical evaluation, and regulatory approval of this dosage form [2, 4, 11, 24, 25].

Mini-tablets can be manufactured using compression, hot-melt extrusion, electrospinning, and 3D printing [15, 26–28]. From a practical standpoint, direct compression, especially with multi-tip punches, is the most desirable approach because it requires minimal additional equipment, entails lower capital investment, and offers high productivity [12, 13, 15, 29]. However, successful compression of mini-tablets in narrow die cavities requires feed blends with sufficiently small particle size, excellent flowability, high bulk density, as well as good compressibility and tabletability to enable die filling without arching [2, 11, 24, 30–34]. The drug loading is another factor that must be considered during formulation development of mini-tablets. Because of their small size, the maximum amount of API that can be incorporated into each unit is limited. Consequently, high to very high drug loading is preferred, particularly for high dose drugs, to reduce the number of mini-tablets per unit dose while still achieving the desired therapeutic response [30, 31, 35]. The reduced number of mini-tablets administered also helps improve patient adherence. However, incorporating a high amount of APIs, especially in micronized form, is challenging because of the intrinsically poor flowability and tabletability of most micron-sized APIs. To address these challenges, numerous approaches have been proposed, including the addition of suitable excipients for direct compression [29, 36, 37], granulation [24, 29, 31, 35, 38], and particle/crystal engineering via milling [30].

The discrete element method (DEM) has become an established numerical tool for investigating the behavior of granular and powder materials under various processing conditions, including mixing, packing, and compaction [39–41]. In powder compaction, DEM provides direct access to particle-scale quantities (e.g., contact forces, force chains, and particle rearrangements) that are difficult to measure experimentally yet strongly influence macroscopic behavior, particularly in narrow die cavities where wall effects and geometric constraints are significant [42–44]. By explicitly resolving particle–particle and particle–tooling interactions, DEM simulations enable systematic studies of how formulation and material variables, including powder weight and particle size distribution, influence force transmission and compaction behavior, particularly under the strong confinement conditions characteristic of mini-tablet tooling [45, 46].

The objective of this study was to systematically investigate how particle size and particle size distribution influence force transmission and compaction behavior of powders confined within narrow die cavities. Using DEM modeling, powder compaction was simulated under controlled conditions to examine both the overall force–displacement response and the forces acting at individual particle contacts. The DEM parameters were optimized using experimental data obtained from a compaction simulator. This work focuses on how changes in powder weight, particle size, and size distribution breadth influence normal, tangential, and cohesive forces during compaction. In addition, the spatial distribution of these forces was analyzed along the axial and radial directions of the die at the point where the upper punch reached its maximum applied force, and particle-scale force networks were visualized using cross-sectional maps. Together, these analyses provide a clearer picture of how load is transmitted through the powder bed and how particle size and polydispersity shape the development of force networks during compaction.

## Materials and Methods

### Materials

Sugar pellets (pharm-a-sphere, Hanns G. Werner, Tornesch, Germany) were used herein. Pellets with particle size of 250 µm were obtained by sieving as-received pellets through 250 μm aperture size (60 mesh) sieve. The pellets that were retained on the mesh were carefully collected for compaction studies.

### Experimental Methods

#### True Density Measurements

Pellet true density was determined at 25 ± 2 °C/40 ± 5% RH by helium pycnometry (Ultrapyc 5000, Antor Paar, Vernon Hills, IL). Prior to measurement, samples were dried in a hot air oven at 70 ºC overnight to remove residual moisture. The target pressure was set at 10 psi. For each sample, 15 runs were performed and the results were averaged.

#### Mini-Tablet Preparation

Compaction was performed using a 2 mm flat-face punches-and-die set (Natoli Engineering, St Charles, MO) mounted on a compaction simulator (Styl’One Evolution, Medelpharm, Beynost, France). The default cycle with one compaction was used, with the compaction rate reduced to 25%. Briefly, the compression, relaxation, and ejection times were 160, 40, and 160 ms, respectively. Machine deformation was corrected using a blank compaction run with 11.28 mm flat-faced punches and die as a reference. Prior to each compaction cycle, the punches and die were lubricated by spraying magnesium stearate suspension (Styl’One Mist, Medelpharm, Beynost, France) which contains 0.3 mg magnesium stearate per spray. The pellets were manually filled into the die. Fill volume was controlled by changing the die fill height. The target force was obtained by changing the thickness of the compacts. A frequency of 1000 Hz was applied to record the force and displacement data using the Analis software (Version 2.08.8, Medelpharm, Beynost, France).

#### Mini-Tablet Characterization

Following ejection, the weight of mini-tablets was determined using a microbalance (Sartorius Lab Instruments, Göttingen, Germany) with a readability interval of d = 0.1 mg. The dimensions of mini-tablets, including thickness and diameter, were measured using a digital thickness gauge (Absolute 547-500S, Mitutoyo Corp., Kanagawa, Japan) with a resolution of 0.01 mm. The density of the mini-tablets was calculated from their weight and volume. The solid fraction of the mini-tablets was defined as the ratio of the mini-tablet density to the true density of the pellets. The breaking force of the mini-tablets was measured using a tablet hardness tester (YD-1, Laboao, Henan, China) with an accuracy of ± 0.5%. Tensile strength (TS) of the mini-tablets was then calculated using the following equation:

1 $$TS=\frac{2F}{\pi dH}$$

where F, d, and H represent the breaking force, diameter, and thickness of the mini-tablets, respectively.

### Computational Methods

A numerical DEM model was developed using Rocky (2025R1 version, Ansys, Canonsburg, PA) to simulate the powder compaction process during tableting. In the DEM framework, the rotational and translational motions of the inelastic, frictional, spherical particles are governed by Newton’s second law and Euler’s law, respectively, which are as follows:

2 $${m}_{i}\frac{d{v}_{i}}{dt}=\sum {F}_{c}+{m}_{i}g$$

3 $${J}_{p}\frac{d{w}_{p}}{dt}=\sum {M}_{c}$$

where ${m}_{i}$ represents the particle mass, ${v}_{i}$ is the particle translational velocity, and g is the gravitational acceleration. ${F}_{c}$ accounts for both particle–particle and particle–wall interactions, and the particle angular velocity is defined by ${w}_{p}$. The particle moment of inertia is ${J}_{p}$, and ${M}_{c}$ represents the net torque arising from tangential contact forces, which induces the rotational motion of the particle.

The contact forces between the particles in this study were modeled using the nonlinear Edinburgh Elasto-Plastic-Adhesive (EEPA) and linear spring–Coulomb limit models, which describe the normal and tangential forces, respectively. A summary of each model is given in Sects. "Normal Force Model" and "Tangential Force Model", and we refer readers to the Ansys Rocky user manual for more detailed information [47].

#### Normal Force Model

The normal force model is as follows:

4 $$F_{EEPA}=\left\{\begin{array}{l}\begin{array}{ccc}F_1=K_1S_n^\frac32&if&F_2\leq F_1\end{array}\\\begin{array}{c}\begin{array}{ccc}F_2=K_2\left(s^\frac32-s_p^\frac32\right)&if&F_1>F_2>F_a\end{array}\\\begin{array}{ccc}F_a=-K_as&if&F_a>F_2\end{array}\end{array}\end{array}\right.$$

This model is based on the classical contact theory proposed by Morrissey [48] in which ${K}_{1}$ and ${K}_{2}$ are the stiffnesses coefficients for loading and unloading, respectively, s is the current overlap, ${S}_{n}$ represents the normal contact overlap, and ${s}_{p}$ denotes plastic deformation. ${K}_{a}$ is the adhesion stiffness, while ${F}_{1}$, ${F}_{2}$, and ${F}_{a}$ are the loading, unloading, and adhesion forces, respectively. Since all particles in this study are composed of the same material, the adhesion force represents cohesive interactions between particles.

#### Tangential Force Model

The tangential force in this study was simulated using a linear spring–Coulomb limit model, which follows an elastic–frictional behavior and constrains the tangential force such that it does not exceed the Coulomb friction limit. This can be expressed as

5 $${F}_{\tau }^{t}=\mathrm{m}\mathrm{i}\mathrm{n}(\left|{F}_{\tau ,e}^{t}\right|,{\mu F}_{n}^{t})\frac{{F}_{\tau ,e}^{t}}{\left|{F}_{\tau ,e}^{t}\right|}$$

In this model, ${F}_{n}^{t}$ represents the contact normal force at time t, μ is the friction coefficient, and ${F}_{\tau }^{t}$ and ${F}_{\tau ,e}^{t}$ are the tangential contact force and elastic tangential force, respectively. All simulations were performed using the University of Connecticut high-performance computing (HPC) facilities, and the simulation parameters used in this study are listed in Table I. A sensitivity analysis is included in Supplementary Information, Appendix A.

**Table I:** Parameters used in DEM simulations with their corresponding values

Simulation parameter	Value
Young modulus (N/m^2^)	4.4 × 10^9^
Poisson ratio	0.3
Restitution coefficient	0.1
Particle–wall and particle–particle friction	0.1
Surface energy (J/m^2^)	1
Density (kg/m^3^)	1565.3
Plasticity ratio	0.99
Damping ratio	0.7
Time step (s)	4.5 × 10^−9^ to 1.1 × 10^−8^

#### Application of a Log-Linear Model to Construct Particle Size Distributions

A log-linear model based on the lognormal cumulative distribution function (CDF) was used to construct the particle size distributions evaluated herein. For each sieve aperture d, the untruncated lognormal CDF was calculated using the closed-form expression:

6 $$F\left(d\right)=\frac{1}{2}\left[1+\mathrm{e}\mathrm{r}\mathrm{f}(\frac{\mathrm{ln}\,d-\mathrm{ln}\,{D}_{50}}{\sigma \sqrt{2}})\right]$$

where ${D}_{50}$ is the median particle diameter and σ is the standard deviation in the logarithmic space. This equation was applied directly to the nominal aperture sizes of the ISO 565 sieve series spanning 212 to 300 µm (narrow), 150 to 425 µm (intermediate), and 90 to 710 µm (wide). The values at the lower and upper bounds of each range, denoted ${F}_{lower}$ and ${F}_{upper}$, respectively, were used to normalize the CDF and enforce truncation. The truncated cumulative distribution was calculated as

7 $${F}_{t}\left(d\right)=\frac{F\left(d\right)-{F}_{lower}}{{F}_{upper}-{F}_{lower}}$$

The transformation applied forces the cumulative probability to be zero at the lower sieve limit and one at the upper sieve limit for each particle size distribution, while preserving the relative shape of the lognormal model within that interval. Incremental mass fractions for each sieve class were obtained from the differences between successive truncated cumulative values. These truncated and interval-based quantities formed the basis of the cumulative and retained particle size distributions used in subsequent analyses.

Within the wide particle size window (90–710 µm), additional distributions were generated by changing the standard deviation to obtain different degrees of breadth while maintaining the same truncation bounds. Three subclasses were defined as W_narrow_ $\left(\sigma=0.25\right)$, W_intermediate_ $\left(\sigma=0.4\right)$, and W_wide_ $\left(\sigma=1\right)$. These distributions share the same lower and upper limits but differ in the spread of particle sizes around the median. The complete particle size distribution tables for each truncation range and σ value are provided in the Supplementary Information (Tables S1–S5).

### Force–Displacement Curve Analysis

Numerical integration of the force–displacement curves obtained from DEM simulations was performed in MATLAB Online (The MathWorks, Inc., Natick, MA) using the trapezoidal rule. The onset of compression was defined as the punch displacement $\left(\delta\right)$ at which the force first exceeded 0.01 N. Forces below this value were attributed to numerical noise and were excluded from the analysis.

The total work input during compression, ${W}_{total}$, was calculated as

8 $${W}_{total}={\int}_{{\delta}_{F\ge 0.01}}^{{\delta}_{max}}{F}_{loading}\left(\delta \right)d\delta$$

where ${\delta}_{F\ge 0.01}$ is the displacement at which the force first exceeds 0.01 N, ${\delta}_{max}$ is the maximum punch displacement, and ${F}_{loading}\left(\delta \right)$ is the force recorded during the loading phase as a function of displacement.

The recoverable elastic work, ${W}_{elastic}$, was obtained from the unloading curve as

9 $${W}_{elastic}={\int}_{{\delta}_{max}}^{{\delta}_{f}}{F}_{unloading}(\delta )d\delta$$

where ${\delta}_{f}$ is the final displacement at which the force returns to approximately zero during unloading, and ${F}_{unloading}(\delta )$ is the force during unloading.

The ideal linear work at maximum displacement was calculated as

10 $${W}_{linear}=\frac{1}{2}{F}_{loading,max}\left({\delta}_{max}-{\delta}_{F\ge 0.01}\right)$$

where ${F}_{loading,max}$ is the peak force measured at ${\delta}_{max}$.

Plastic energy (PE) was computed as

11 $${PE=W}_{total}-W_{elastic}$$

and rearrangement energy (RE) was computed as

12 $$RE={W}_{linear}-{W}_{total}$$

These values were used to calculate the RE/PE ratio.

## Results

### Mini-Tablet Attributes

Mini-tablets exhibited a uniform appearance, with relatively smooth surfaces and no visible defects. Weight uniformity was acceptable, with relative standard deviation values of 2.18% and 2.87% for six consecutive runs at fill depths of 4 mm and 5 mm, respectively. The solid fraction of the mini-tablets was measured both in-die at maximum compression force and out-of-die after elastic recovery. For mini-tablets produced with a 4 mm fill depth, the in-die and out-of-die solid fractions were 0.91 ± 0.02 and 0.84 ± 0.02, respectively. Corresponding values for mini-tablets with a 5 mm fill depth were 0.89 ± 0.02 and 0.82 ± 0.02. The fill depths of 4 mm and 5 mm resulted in mini-tablets with tensile strength of 0.18 ± 0.05 MPa and 0.12 ± 0.03 MPa, respectively.

### Model Validation

**Fig. 1:**
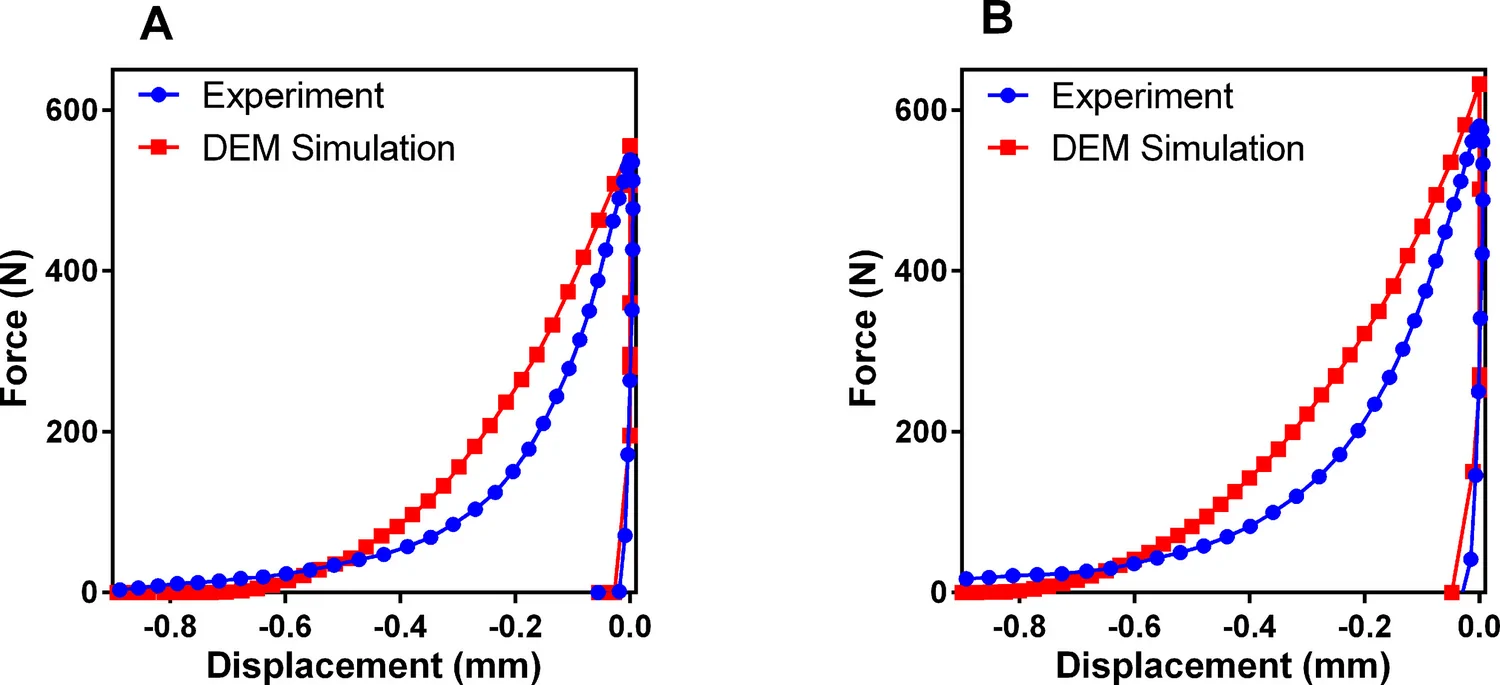
Comparison of upper punch force–displacement profiles obtained from DEM simulations and experiments, showing very good agreement. The fill depths were (**A**) 4 mm and (**B**) 5 mm.

Two sets of experiments were selected to validate the model and simulation parameters. The normal force experienced by the upper punch during compression as a function of normalized punch displacement, as obtained from both experiments and DEM simulations, is shown in Fig. 1. In both cases, spherical particles with a diameter of 250 µm were used. The fill depths were 4 mm (Fig. 1A) and 5 mm (Fig. 1B), respectively, and in both cases, compression was performed to a final height of 2 mm. As illustrated, the simulated trends are in good agreement with the experimental results. During the early stages of compression, powder particles could rearrange to fill the void spaces, requiring relatively low forces. As compression continued, the particles became denser and offered greater resistance to further compression, with the required force increasing sharply at higher displacements.

Across the entire displacement range examined, the DEM simulation data closely aligned with the experimental measurements. The minor differences between experimental and simulated force–displacement profiles can be attributed to inherent simplifications in the numerical representation of the material. Although the EEPA contact model accounts for elasto-plastic deformation and cohesion, the simulations assume mechanically identical spherical particles without internal porosity or localized crushing. In contrast, sugar pellets possess slight shape irregularities, internal structural heterogeneity, and a narrow but finite size distribution even after sieving. These differences may contribute to the small deviations observed at higher compression forces.

### Effect of Powder Fill Weight

**Fig. 2:**
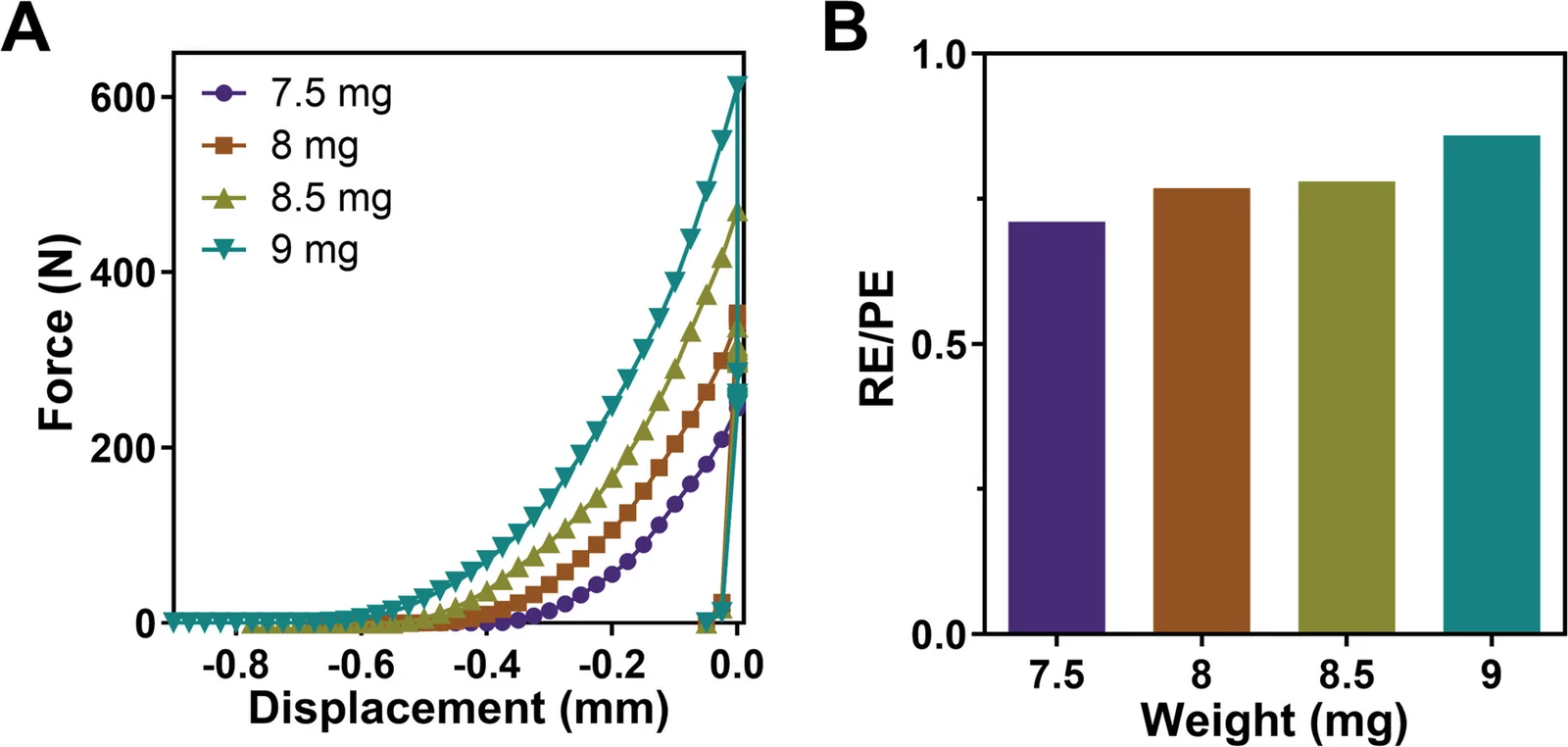
(**A**) At a fixed displacement, higher fill weights corresponded to larger maximum forces. This translated to (**B**) increased RE/PE ratio.

The force–displacement curves for fill weights of 7.5, 8, 8.5, and 9 mg showed that the force remained near zero during the initial stages of compression and then increased sharply as the punch approached the final displacement (Fig. 2A). As powder weight increased, the force profiles trended upward. The 7.5 mg sample exhibited the lowest peak force at the end of compression, while the 9 mg sample showed the highest peak force, exceeding 600 N. Higher fill weights also resulted in more energy expended as rearrangement energy during powder consolidation, as reflected by the increase in RE/PE ratio (Fig. 2B).

#### Axial Force Distributions

**Fig. 3:**
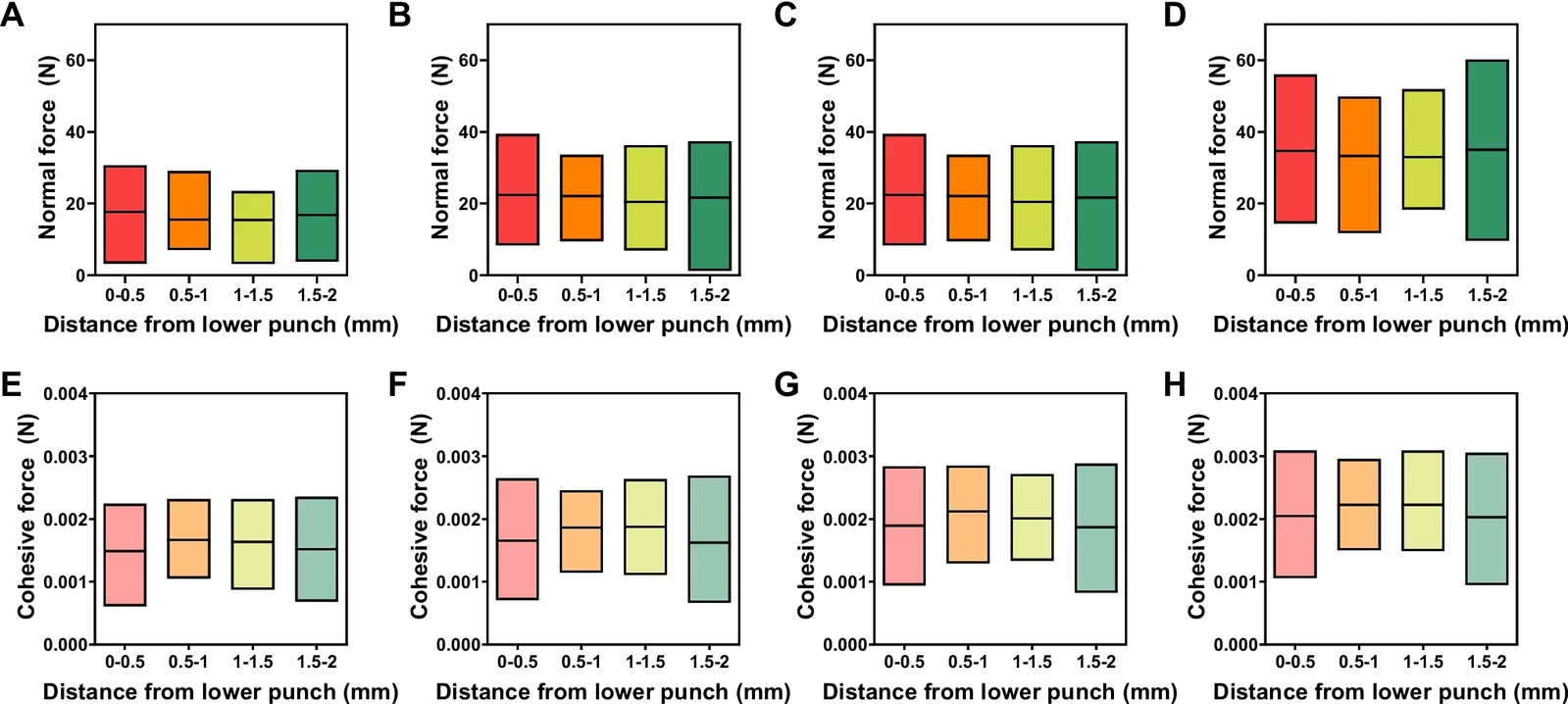
Axial normal force distributions for (**A**) 7.5 mg, (**B**) 8 mg, (**C**) 8.5 mg, and (**D**) 9 mg samples showed a systematic increase in median force with increasing tablet weight. Axial cohesive force distributions for (**E**) 7.5 mg, (**F**) 8 mg, (**G**) 8.5 mg, and (**H**) 9 mg remained relatively uniform across Z-segments.

Axially, normal and tangential forces spanned a wide range across the powder bed (Fig. 3A–D, Figure S1A–D). The median normal and tangential forces were slightly reduced in the intermediate layers (0.5 to 1.5 mm from the lower punch), whereas the top and bottom layers showed greater variability, suggesting that boundary effects influenced local force transmission. In contrast, axial cohesive forces were relatively uniform across the four Z-segments (Fig. 3E–H), with slightly reduced values near the lower and upper punch compared to the tablet core. These observations highlighted contrasting spatial patterns, where normal and tangential forces exhibited pronounced boundary effects, while cohesive forces remained relatively consistent except for modest reductions at the boundaries.

#### Radial Force Distributions

**Fig. 4:**
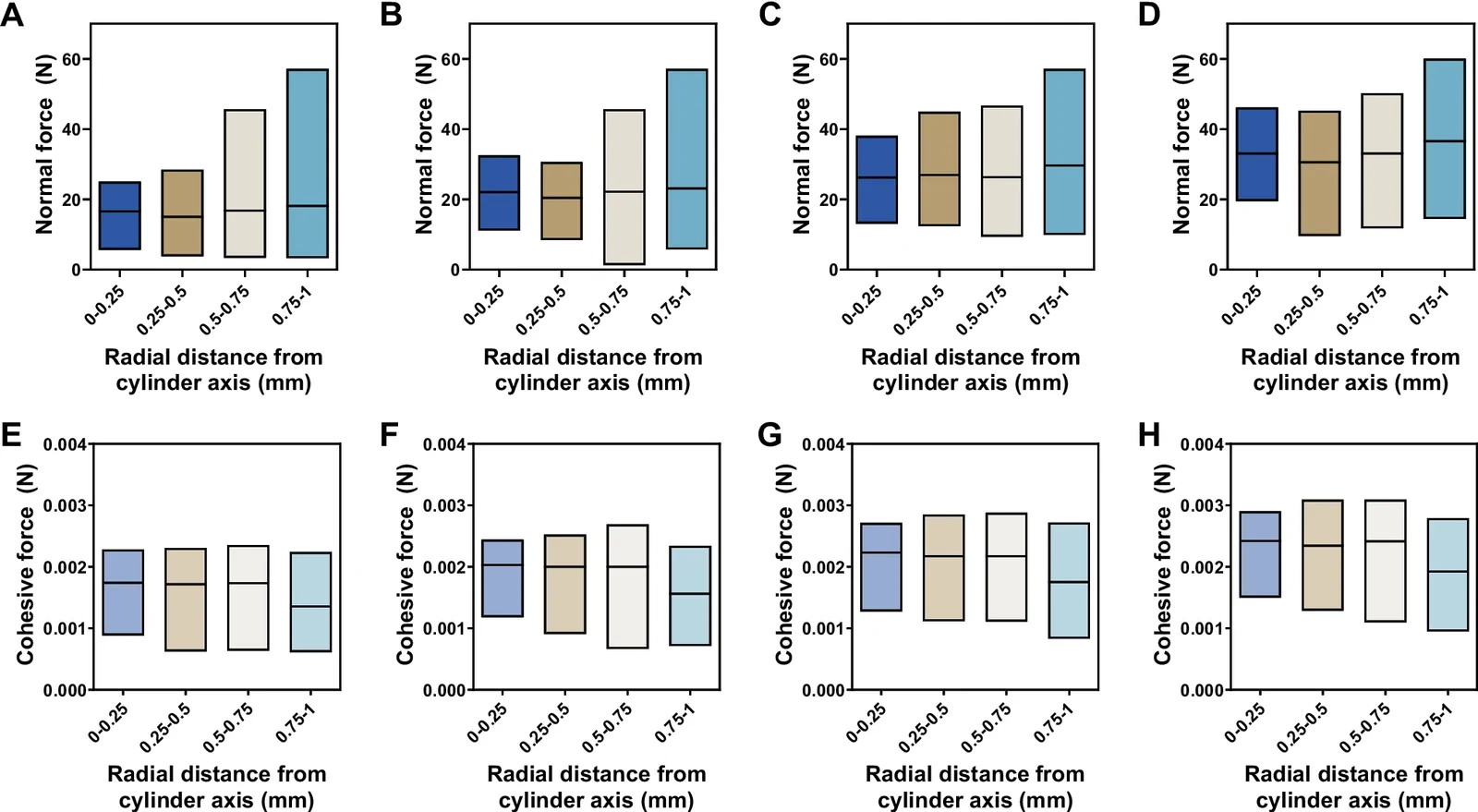
Radial normal force distributions for (**A**) 7.5 mg, (**B**) 8 mg, (**C**) 8.5 mg, and (**D**) 9 mg samples showed a systematic increase in median force with increasing weight. Radial cohesive force distributions for (**E**) 7.5 mg, (**F**) 8 mg, (**G**) 8.5 mg, and (**H**) 9 mg exhibited a decrease in force magnitude with radial distance from the cylinder axis, with a substantial drop in median force observed for particles closest to the radial die wall.

Radially, normal and tangential forces increased steadily from the cylinder axis toward the die wall (Fig. 4A–D, Figure S1E–H). Median normal force values remained relatively similar across radial positions, but variability was the smallest near the axis (0 to 0.25 mm) and largest near the outermost region (0.75 to 1 mm), where maximum normal force exceeded 60 N. This trend was most evident for the 7.5 mg and 8.0 mg samples, where particles adjacent to the die wall displayed substantially greater variability than those near the axis. In contrast, radial cohesive forces exhibited an opposite pattern: values decreased progressively with radial distance, with the lowest medians observed near the die wall (Fig. 4E–H). These observations indicated that lateral confinement and wall-supported stress transfer strongly influenced local force distributions, while particle packing and sample weight governed overall force magnitude and variability within the powder bed.

#### Particle-Level Visualizations

Although increasing fill weight increased the peak compaction force, the overall topology of force transmission within the compact remained qualitatively similar across weights. In all cases, force transmission occurred through localized contact networks that intensified with increasing weight, suggesting that fill weight primarily scaled the magnitude of the applied load rather than fundamentally altering the underlying force network structure.

**Fig. 5:**
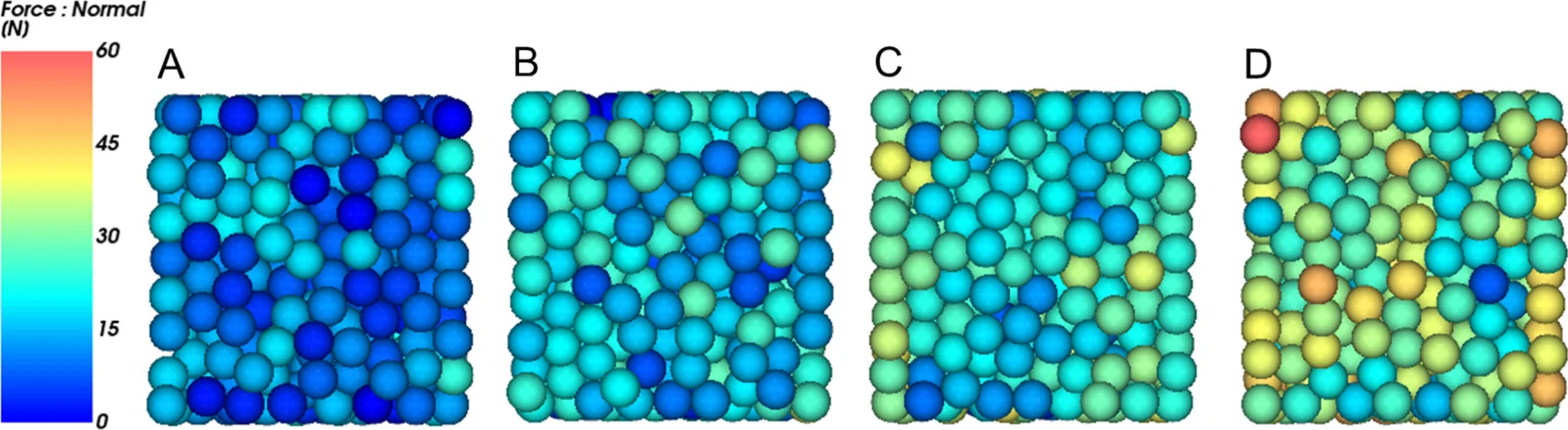
Particle-level normal force distributions within a compact cross section at (**A**) 7.5 mg, (**B**) 8 mg, (**C**) 8.5 mg, and (**D**) 9 mg. Colors indicate the magnitude of the normal force acting on individual particles, from low (blue) to high (red). High-force clusters formed continuous paths toward the die wall, indicative of force chains that intensified with increasing tablet weight.

**Fig. 6:**
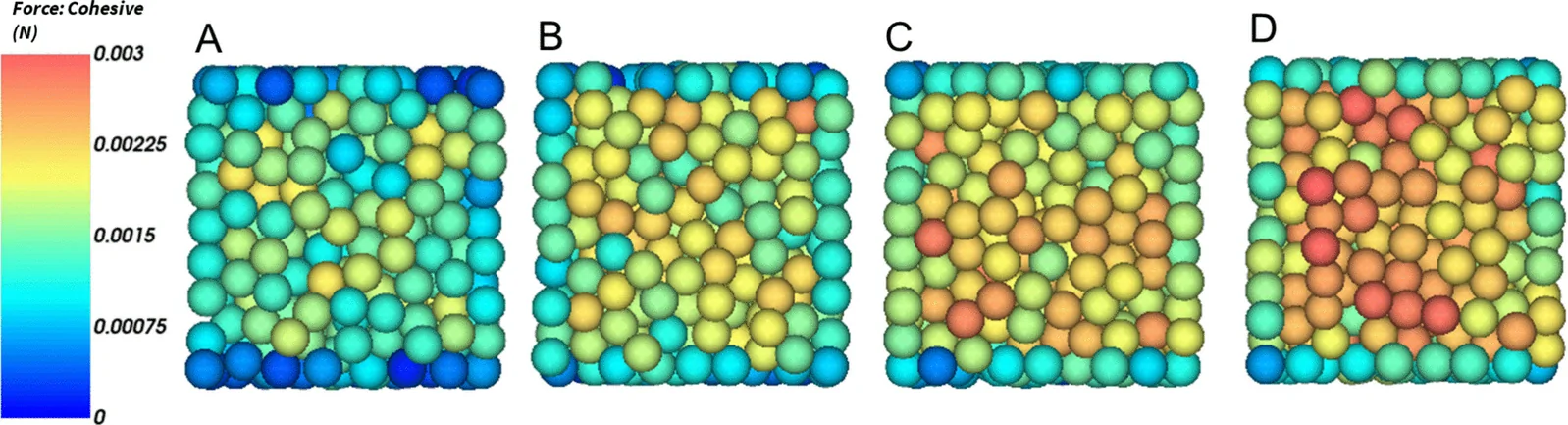
Particle-level cohesive force distributions within a compact cross section at (**A**) 7.5 mg, (**B**) 8 mg, (**C**) 8.5 mg, and (**D**) 9 mg. Cohesive forces remained relatively uniform across the bed but decreased near the die wall, forming an outer band of reduced bonding. Higher weights produced larger regions of elevated cohesive forces in the center of the compact.

Particle-level maps revealed distinct spatial organization of forces within the compact (Figs. 5 and 6). For normal forces, localized clusters of high-magnitude contacts formed continuous paths extending toward the die wall, particularly at higher tablet weights. At 7.5 mg, most particles experienced low to moderate normal forces, with only isolated high-force contacts (Fig. 5A). At 9 mg, the compact displayed the broadest range of normal forces, and localized clusters of high-force particles formed continuous paths toward the die wall (Fig. 5D). These force chains represented preferential load-bearing networks that intensified with increasing tablet weight, reflecting the role of lateral confinement in stress transmission.

Tangential force distributions similarly displayed localized high-force regions near the die wall, indicating frictional interactions along lateral boundaries. At 7.5 mg, tangential forces were uniformly low, with only a few isolated moderate-force contacts (Figure S2A). As the powder weight increased to 8 and 8.5 mg, these high-force clusters became more frequent and expanded along the lateral boundaries. At 9 mg, the compact contained the largest number of high-force clusters concentrated near the die wall, with additional scattered particles in the interior experiencing moderate-to-high tangential forces, indicating that frictional interactions extended inward under greater confinement (Figure S2D). Unlike normal forces, which formed continuous chains, tangential forces organized into discrete clusters that grew in both size and intensity with increasing tablet weight.

Cross-sectional views revealed a clear increase in cohesive forces within the compact interior as powder weight increased from 7.5 mg to 9.0 mg. At lower weights, cohesion was weak and relatively uniform, whereas higher weights produced progressively larger regions of elevated cohesive forces, especially in the central zones (Fig. 6). Across all four cases, particles adjacent to the die wall consistently exhibited the lowest cohesive forces, forming a distinct outer band of reduced bonding. In contrast, the interior particles showed a sharp rise in cohesive force with increasing weight, highlighting the combined influence of particle packing and confinement on interparticle bonding.

### Effect of Particle Size in Monodisperse Systems

**Fig. 7:**
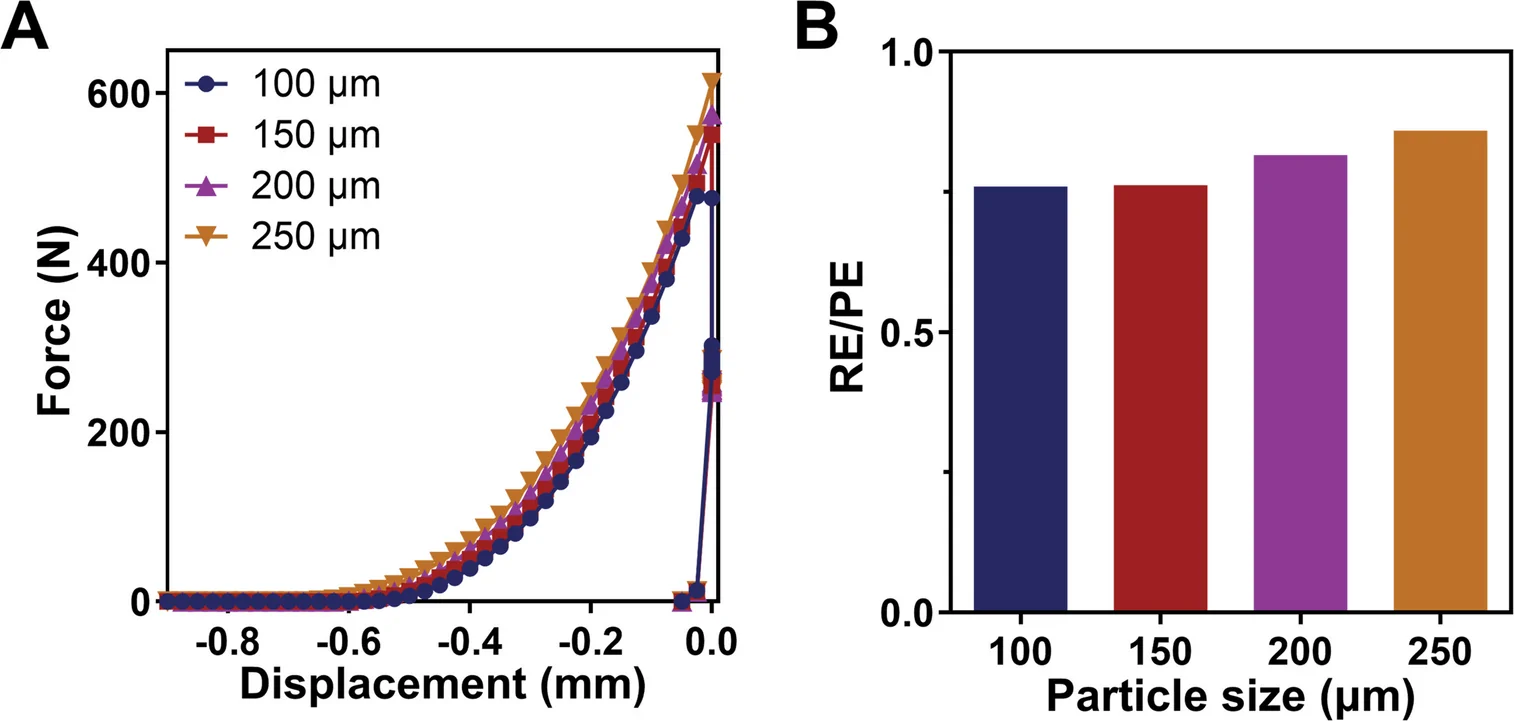
(**A**) Force–displacement profiles showed increasing force at a given displacement with increasing particle size, with larger particles producing higher maximum forces. This trend translated to (**B**) increased RE/PE ratio.

The force–displacement curves for particle sizes of 100, 150, 200, and 250 µm at 9 mg fill weight are shown in Fig. 7A. All the curves followed a similar nonlinear force increase as displacement approached the pre-determined tablet thickness, but the peak force at the end of compression rose with increasing particle size. The maximum force recorded was the lowest for the 100 µm particles, while the 250 µm particles generated the highest force, reaching slightly above 600 N. Differences were more evident in the RE/PE ratios (Fig. 7B), which showed a progressive increase with increasing particle size [49].

#### Axial Force Distributions

**Fig. 8:**
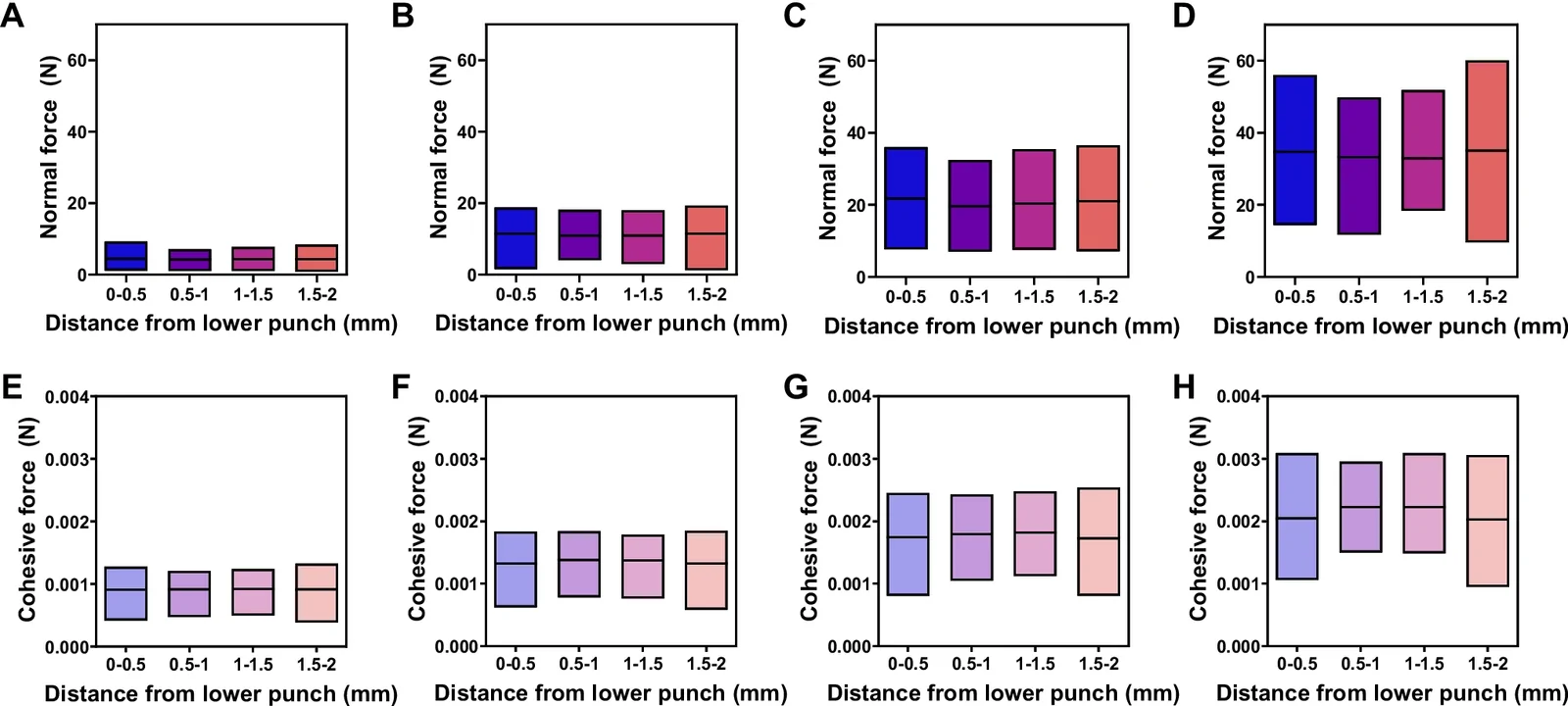
Axial normal force distributions for (**A**) 100 µm, (**B**) 150 µm, (**C**) 200 µm, and (**D**) 250 µm samples showed a systematic increase in median force with increasing particle size. Axial cohesive force distributions for (**E**) 100 µm, (**F**) 150 µm, (**G**) 200 µm, and (**H**) 250 µm also showed increasing median with increase in particle size.

Figure 8 shows the axial distributions of particle-level normal and cohesive forces for particle sizes ranging from 100 to 250 µm. Across all particle sizes, the median axial normal force increased systematically with increasing particle size, which indicates enhanced load transmission through particle–particle contacts. Larger particles also exhibited broader force distributions, suggesting increased variability in axial force transmission. With fewer load-bearing contacts available, stress became concentrated along limited transmission pathways, resulting in pronounced force chains. In contrast, the 100 µm and 150 µm samples showed narrower distributions (Fig. 8A and B). Within each particle size, normal forces were relatively uniform along the axial direction, with only modest boundary effects near the punches. Tangential force distributions (Figure S3A–D) followed the same trends as normal forces, including the increase in both median force and distribution width with particle size. These observations indicated that particle size influenced not only the magnitude but also the heterogeneity of axial force transmission within the compact. Axial cohesive forces displayed significantly lower magnitudes, but similarly showed increasing distribution width with particle size, while smaller particles maintained more uniform cohesive force distributions (Fig. 8E–H).

#### Radial Force Distributions

**Fig. 9:**
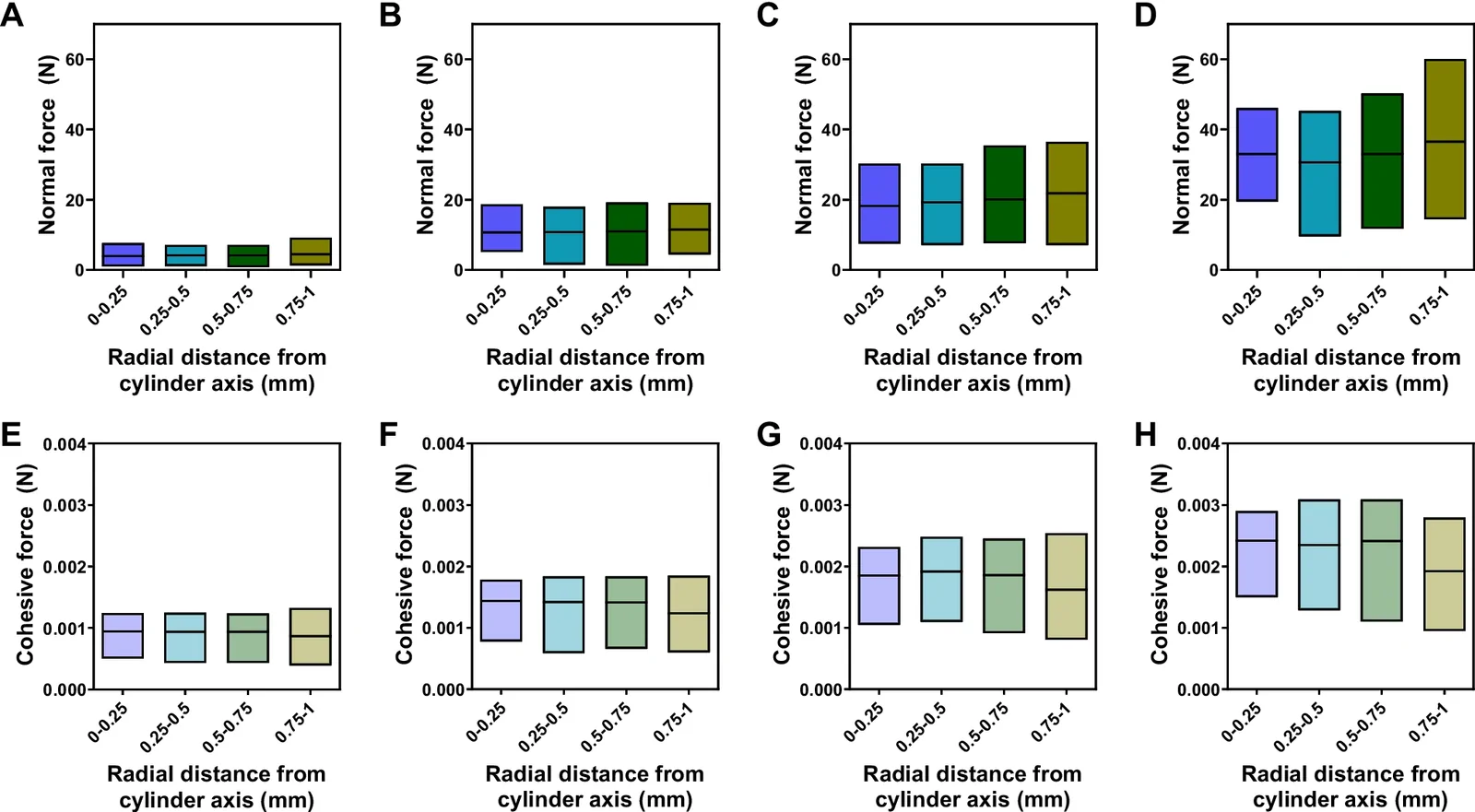
Radial normal force distributions for (**A**) 100 µm, (**B**) 150 µm, (**C**) 200 µm, and (**D**) 250 µm samples showed a systematic increase in median force with increasing particle size. Radial cohesive force distributions for (**E**) 100 µm, (**F**) 150 µm, (**G**) 200 µm, and (**H**) 250 µm particles exhibited a substantial drop in median force for particles closest to the radial die wall.

Radially, increase in particle size corresponded to an increase in both normal and tangential forces (Fig. 9A–D, Figure S3E–H). Similar to the trend observed with axial force distributions, particles located adjacent to the die wall consistently exhibited the highest median normal and tangential forces, as well as the largest spread. The exception to this trend was the 250 µm sample, where particles within the central radial section exhibited a slightly higher median normal and tangential forces (Fig. 9D, Figure S3H). The cohesive force distributions exhibited a similar pattern (Fig. 9E–H). The median cohesive forces consistently decreased as the particle size reduced from 250 µm to 100 µm. As observed for the axial force distributions, the radial cohesive force distribution was largely uniform across particle sizes, with no pronounced gradient from the cylinder axis to the die wall. A slight reduction in the median cohesive force was, however, observed in the outermost layer adjacent to the die wall.

#### Particle-Level Visualizations

**Fig. 10:**
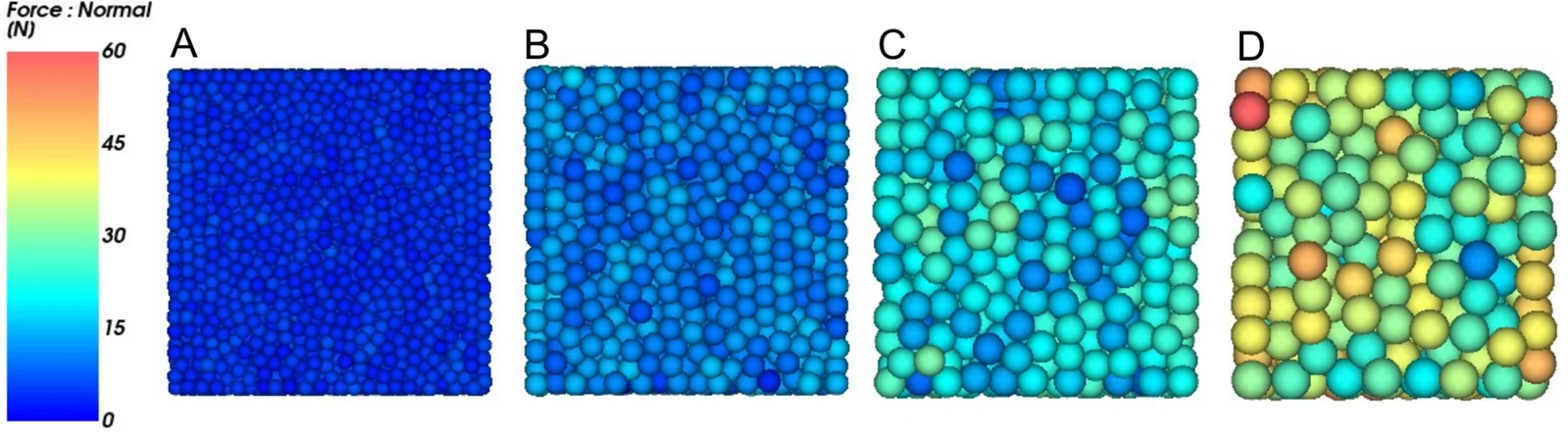
Particle-level normal force distributions within a compact cross section at (**A**) 100 µm, (**B**) 150 µm, (**C**) 200 µm, and (**D**) 250 µm. Colors indicate the magnitude of the normal force acting on individual particles, from low (blue) to high (red).

Particle-level visualization of the normal force distributions provides additional insight into the observed radial and axial force heterogeneity with changes in particle size (Fig. 10). With 250 µm particles, many particles exhibited moderate to high normal forces that appeared as green, yellow, and occasional red regions. In this sample, the highest forces were concentrated in the central region of the cross section, consistent with localized load transmission through a subset of particle contacts. When the particle size decreased to 200 µm, the force field became more uniform and shifted toward lower values, although pockets of moderate force contacts were still present. At 150 µm, the cross section showed predominantly low force contacts, with only scattered regions of slightly elevated forces. For the smallest particle size of 100 µm, the powder bed was dominated almost entirely by low force contacts, shown by the uniform blue coloration across the compact. Overall, the cross-sectional maps demonstrated that both the magnitude and heterogeneity of normal forces decreased noticeably as particle size became smaller, indicating that the applied axial load was distributed more evenly across a larger number of contacts. Although the load was applied axially (top-to-bottom), the normal force distribution in the cross sections appeared more strongly organized radially rather than axially. The emergence of a radially organized force pattern under purely axial applied loading highlights the dominant role of lateral confinement in narrow dies, where wall friction redistributes stresses throughout the compact.

In contrast to the normal force, the tangential force distribution remained comparatively low in magnitude (Figure S4). While larger particles exhibited slightly more variability in tangential forces, localized regions of elevated tangential force were less pronounced than those observed for normal forces, suggesting limited mobilization of frictional slip at individual contacts. As particle size decreased to 150 µm and 100 µm, tangential forces became increasingly uniform, consistent with reduced interparticle sliding and more stable contact configurations. This behavior suggests that, at smaller particle sizes, particle rearrangement was minimized and frictional forces were distributed over many contacts rather than concentrated along distinct force chains, reinforcing the overall homogenization of the force network observed in the normal force field.

**Fig. 11:**
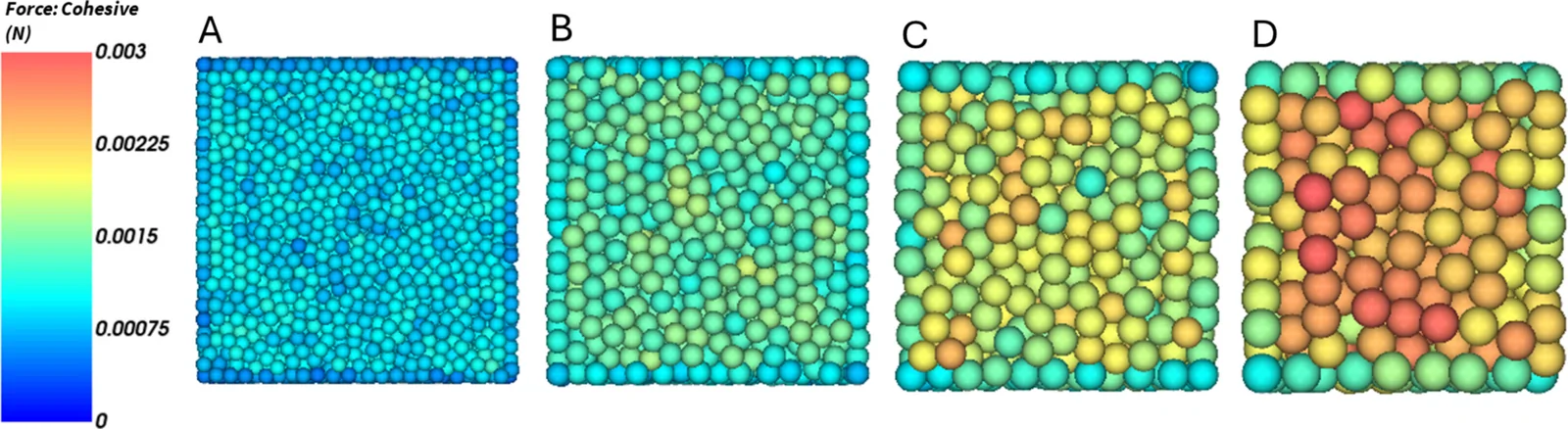
Particle-level cohesive force distributions within a compact cross section at (**A**) 100 µm, (**B**) 150 µm, (**C**) 200 µm, and (**D**) 250 µm. Colors indicate the magnitude of the cohesive force acting on individual particles, from low (blue) to high (red).

Particle-level maps of cohesive force revealed a consistent axial dependence across all particle sizes (Fig. 11). In all compacts, the top and bottom layers exhibited the lowest cohesive forces, indicating weakened cohesive interactions at the loading boundaries. In contrast, particles adjacent to the die wall showed reduced cohesive forces relative to the interior but do not reach the same low levels observed at the top and bottom surfaces. For larger particles (200 µm and 250 µm), the interior region exhibited moderate spatial variability in cohesive forces, whereas decreasing particle size to 150 µm and 100 µm led to a more uniform and generally lower cohesive force distribution. Overall, cohesive force heterogeneity was governed by a combination of particle size effects and boundary-induced axial gradients, with the weakest cohesive interactions localized at the top and bottom of the compact rather than at the die wall.

### Effect of Particle Size Distribution in Polydisperse Systems

**Fig. 12:**
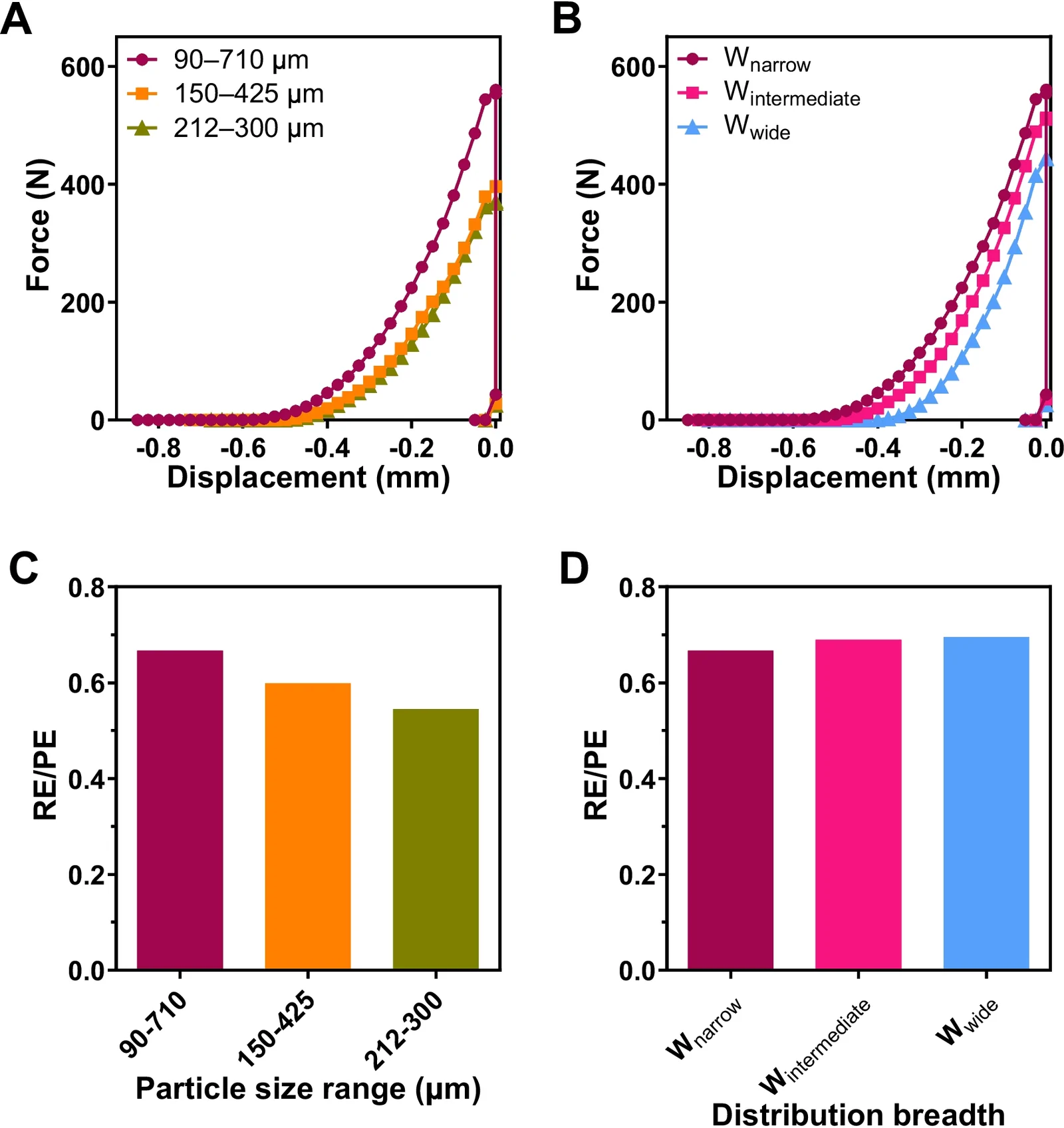
Compression force as a function of punch displacement for powders with different particle size characteristics. (**A**) Effect of particle size distribution, comparing wide (90–710 µm), intermediate (150–425 µm), and narrow (212–300 µm) size distributions. (**B**) Effect of particle size distribution width within the 90–710 µm size range, showing narrow (W_narrow_), intermediate (W_intermediate_), and wide (W_wide_) particle size distributions. (**C**) The RE/PE ratio as a function of particle size range. (**D**) The RE/PE ratio as a function of distribution breadth (W_narrow_, W_intermediate_, W_wide_).

The powder with the widest particle size distribution, 90–710 µm, consistently required the highest compression force for any given punch displacement, reaching the highest peak force of ~ 560 N at minimum distance between the upper and lower punches (Fig. 12A). As the particle size range narrowed to 150–425 µm and further to 212–300 µm, the compression force required to achieve the same displacement decreased significantly, indicating reduced resistance to densification with narrowing size distribution. This observation was further supported by the lower RE/PE ratio as particle size range narrowed, which indicated that particle rearrangement required less work and consolidation proceeded more efficiently (Fig. 12C).

Within the 90–710 µm size window, distribution breadth influenced the macroscopic compaction response (Fig. 12B), even though the RE/PE ratio changed only modestly across the three samples (Fig. 12D). W_narrow_ had the highest compression force across the entire displacement range while W_wide_ consistently required the lowest force to reach the same punch displacement. However, the RE/PE ratio for W_wide_ was also the highest, which suggests that although this sample required the lowest compression force, rearrangement-related processes accounted for a slightly larger share of the total compaction work compared with the narrower distributions.

**Fig. 13:**
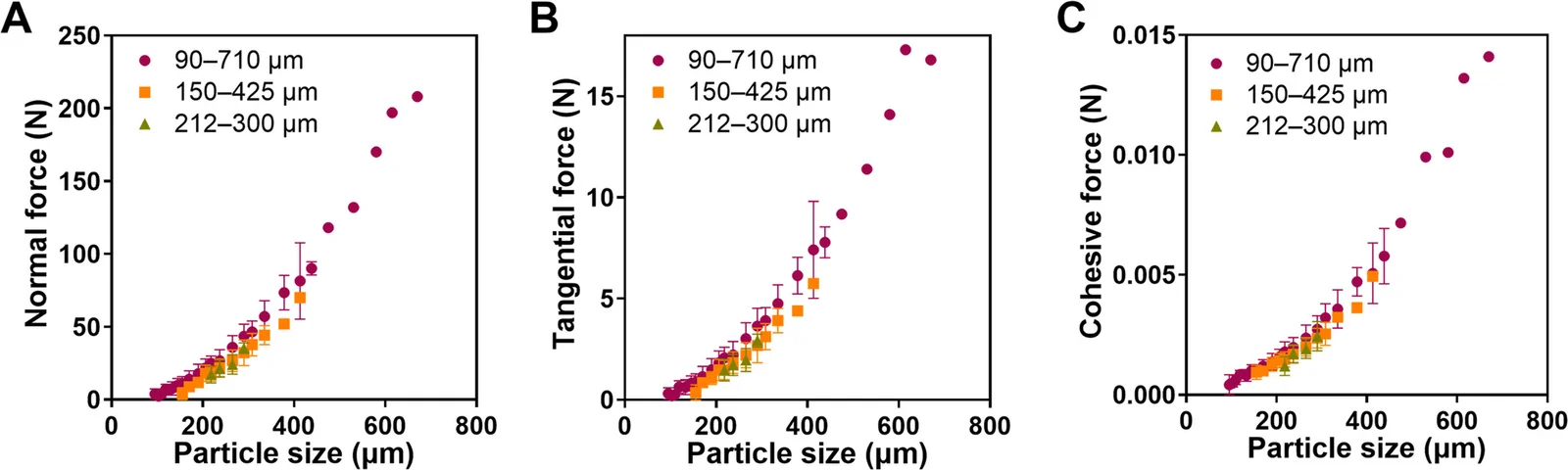
Interparticle force magnitude as a function of particle size obtained from DEM simulations: (**A**) normal force, (**B**) tangential force, and (**C**) cohesive force. Symbols denote mean force values, and error bars indicate the standard deviation across particle contacts. The normal, tangential, and cohesive forces all increased non-linearly with particle size. The largest particles (90–710 µm) generated the highest forces, while the narrowest range (212–300 µm) showed significantly lower magnitudes across all categories.

Across all three particle size ranges, the magnitude of the interparticle force increased with increasing particle size (Fig. 13). For the widest particle size range of 90–710 µm, the maximum normal, tangential, and cohesive forces reached approximately 200 N, 15 N, and 0.015 N, respectively, with all forces increasing continuously as particle size approached 710 µm. This behavior indicates a strong, nonlinear dependence of interparticle force magnitude on particle size. As the particle size range narrowed, the maximum observed forces decreased proportionally. For the intermediate range of 150–425 µm, the peak normal, tangential, and cohesive forces were approximately 75 N, 6 N, and 0.005 N, respectively. The narrowest particle size range of 212–300 µm exhibited the lowest force magnitudes, with peak values of approximately 35 N (normal), 3.5 N (tangential), and 0.0025 N (cohesive).

**Fig. 14:**
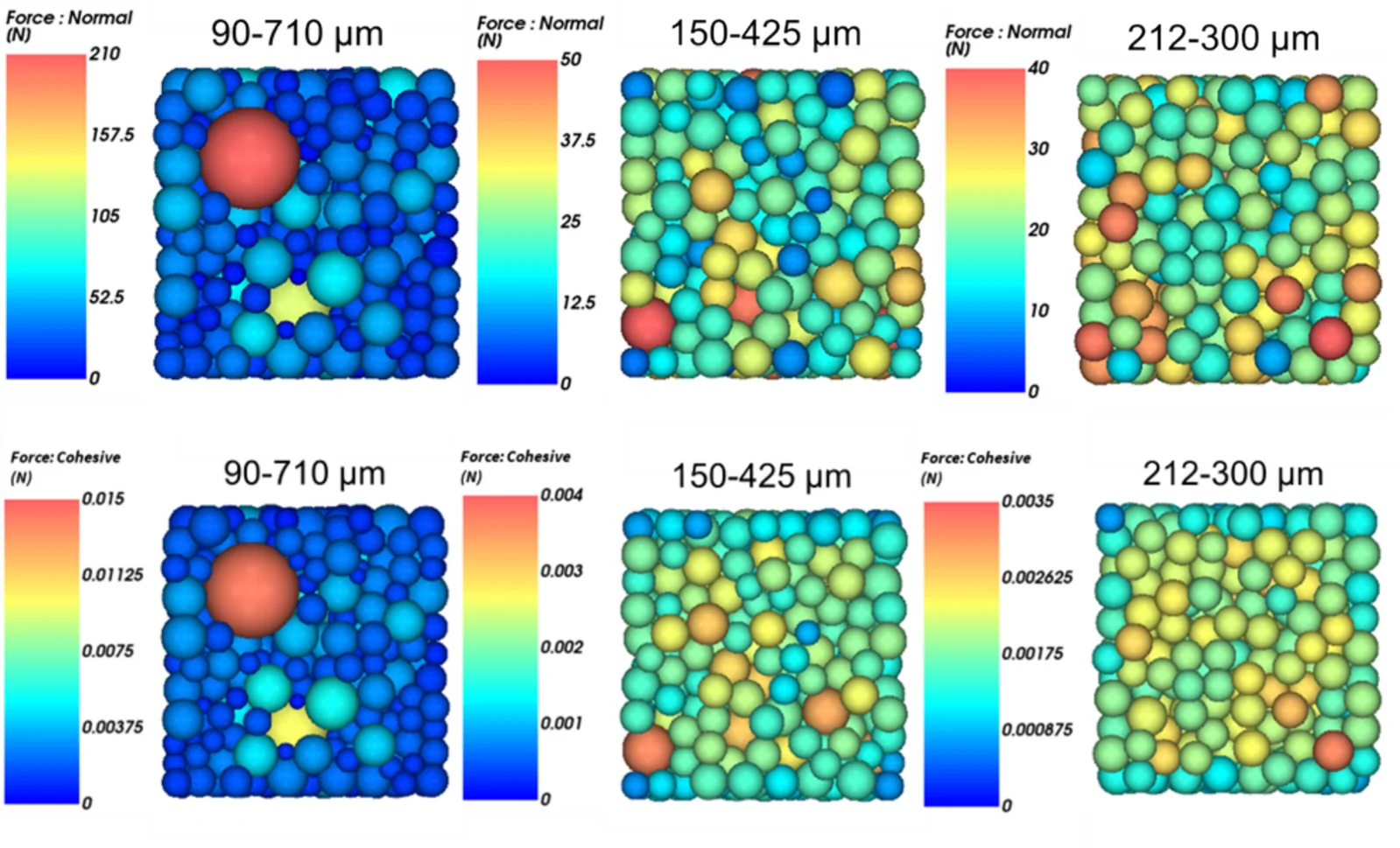
Particle-level normal (top row) and cohesive (bottom row) force distributions within a compact cross section are shown for particle size distributions of 90–710 µm, 150–425 µm, and 212–300 µm. Colors indicate the magnitude of the normal and cohesive forces acting on individual particles, from low (blue) to high (red). For wide (90–710 µm) distribution, a few large particles carried the bulk of the load, forming distinct force chains. In the narrower size ranges, the force network was more collaborative, with forces distributed more evenly across many particles

The particle-level maps revealed the heterogeneity of force networks as a function of particle size distribution. In the widest size range (90–710 µm), the force distribution was highly localized and heterogeneous. As shown in Fig. 14, normal forces concentrated predominantly at contacts involving the largest particles, with one prominent contact exhibiting forces exceeding 100 N (red), while the majority of the surrounding particles experienced forces below 52.5 N (blue). This created a distinct force chain structure where load transmission occurred through a limited number of large particle contacts. The tangential and cohesive force distributions (Figure S5 and Fig. 14) mirrored this pattern, with peak tangential and cohesive forces localized at the same large particle contacts.

For the intermediate and narrow size ranges, the force network became more distributed, as indicated by the reduced visual intensity and more uniform coloration of the force maps. The normal, tangential, and cohesive forces showed a broader spectrum of intermediate values across the powder bed, with multiple particles participating in load transmission rather than single dominant contacts. These trends observed across the three particle size distributions demonstrated that the largest particles in a distribution were the primary drivers of all three types of interparticle forces, creating localized force concentrations that dominate the mechanical behavior of polydisperse particle assemblies.

### Effect of Distribution Breadth within the 90–710 µm Size Window

**Fig. 15:**
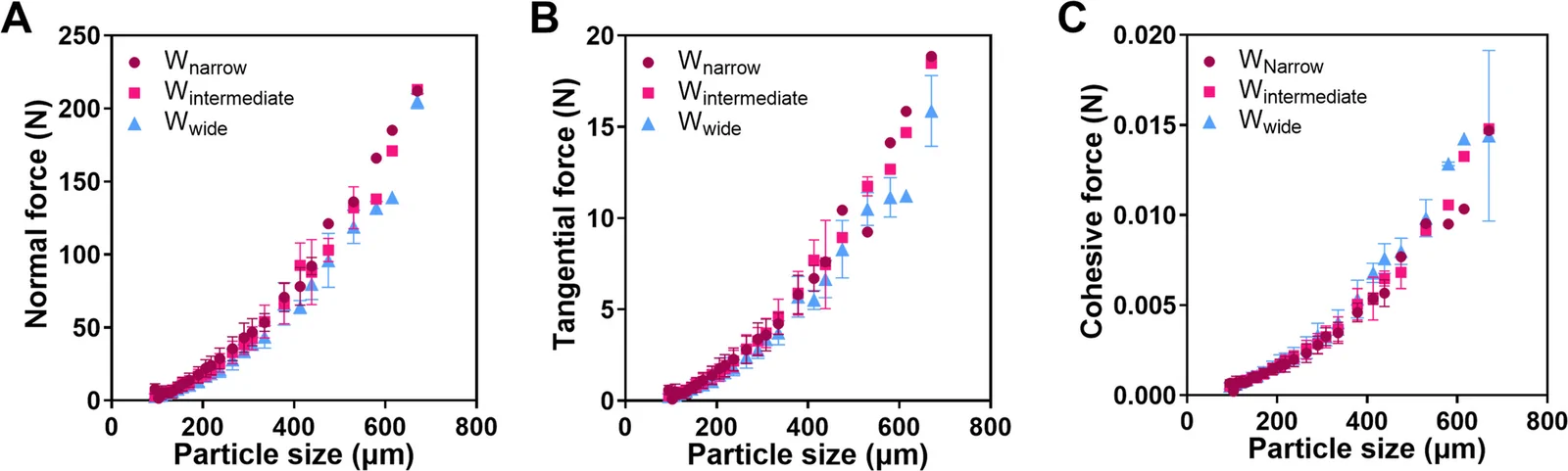
Interparticle force magnitude as a function of particle size obtained from DEM simulations: (**A**) normal force, (**B**) tangential force, and (**C**) cohesive force. Symbols denote mean force values, and error bars indicate the standard deviation across particle contacts. All force types increased with particle size. Narrower distributions created higher normal and tangential forces while wider distributions had higher cohesive forces across most of the size ranges.

Within the 90–710 µm size window, the normal, tangential, and cohesive forces similarly scaled with particle size regardless of the particle distribution breadth, where force increased with increasing particle size (Fig. 15), but the effect of distribution breadth differed between force types.

For normal and tangential forces, narrower distributions consistently exhibited slightly higher force magnitudes compared to wider distributions, particularly at larger particle sizes (> 400 µm). W_narrow_ reached maximum normal forces of approximately 220 N, while W_wide_ peaked around 200 N (Fig. 15A). A similar ordering was observed for the tangential force, where tangential forces for W_narrow_ approached 18 N for the largest particles, compared to approximately 15 N for W_wide_ (Fig. 15B). This trend suggests that narrower distributions may generate more intense force concentrations at particle contacts. Cohesive forces displayed the opposite trend. W_wide_ showed higher cohesive forces than W_narrow_ across most of the particle size range, with all three distributions converging to similar maximum values (approximately 0.014–0.015 N) at the largest particle sizes (Fig. 15C).

**Fig. 16:**
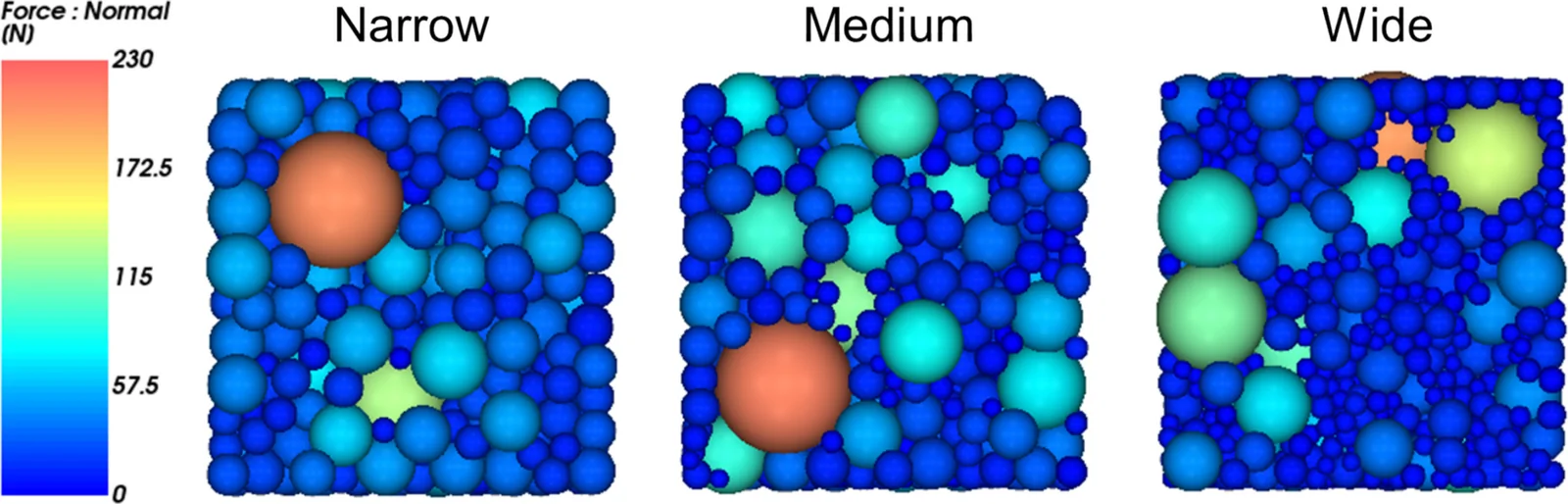
Particle-level normal force distributions within a compact cross section for W_narrow_, W_intermediate_, and W_wide_ within the 90–710 µm size window. Colors indicate the magnitude of the normal force acting on individual particles, from low (blue) to high (red). In the narrow distribution, the force network was highly localized, with a single large particle (red) bearing nearly the entire load. As the distribution widened, greater number of large particles participated in sharing the load across the powder bed

Figures 16, S6, and 17 showed the cross-sectional views of particle-level normal, tangential, and cohesive forces for W_narrow_, W_intermediate_, and W_wide_ within the 90–710 µm size window. In all three force maps, high force particles appear as yellow to red regions and are primarily associated with larger particles, while smaller particles are mostly subjected to low forces shown in blue and cyan.

**Fig. 17:**
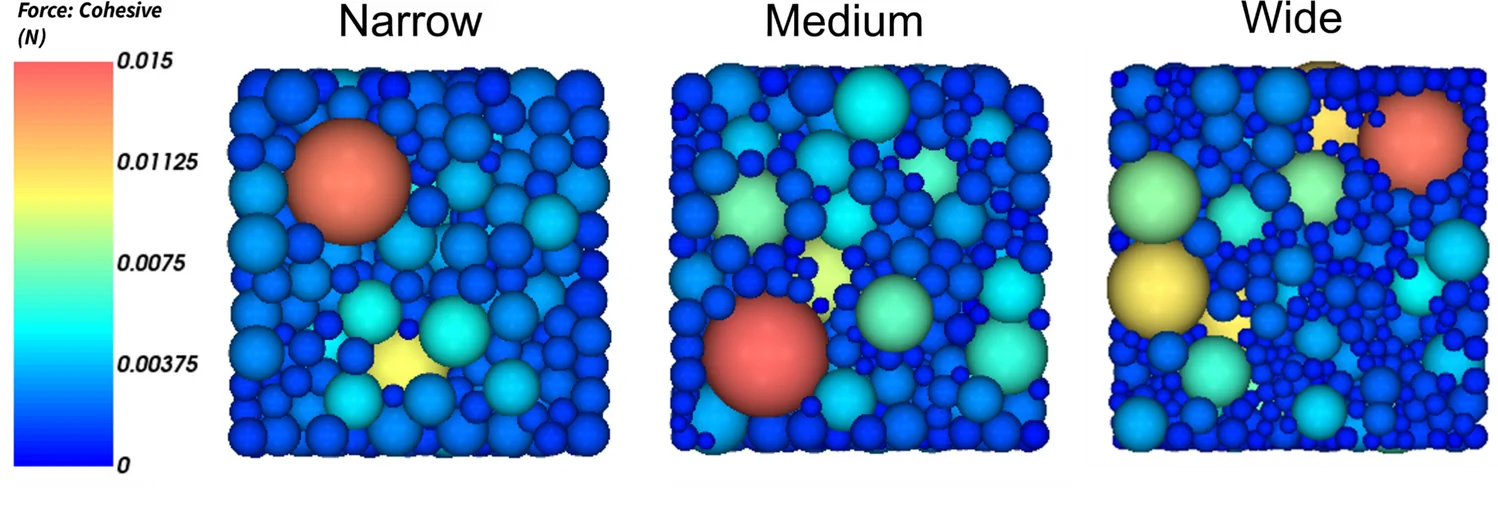
Particle-level cohesive force distributions within a compact cross section for W_narrow_, W_intermediate_, and W_wide_ within the 90–710 µm size window. Colors indicate the magnitude of the cohesive force acting on individual particles, from low (blue) to high (red). While the narrow distribution limits cohesion to a single dominant contact, the wide distribution showed a much broader and dispersed force network across particles

The force maps revealed distinct structural differences in how forces were distributed throughout the powder beds as distribution breadth increased. For W_narrow_, forces were highly localized at a single large particle contact, with one prominent particle (shown in red) dominating the force network for all three force types, while surrounding smaller particles experienced minimal forces (predominantly blue). As distribution breadth increased to intermediate (W_intermediate_), the force network became more distributed, with multiple medium-to-large particles (cyan to yellow-green) participating in load transmission, and forces spreading across several contact points throughout the visible cross-section. The wide distribution (W_wide_) exhibited the most dispersed force network, with multiple large particles of comparable size visible in the cross-section sharing the mechanical load more evenly, as evidenced by several particles displaying intermediate to high force values (cyan to yellow to orange). Notably, for cohesive forces, the wide distribution showed more particles with elevated cohesive forces (yellow to orange coloration) compared to the narrow distribution, supporting the quantitative observation that broader distributions enhance cohesive interactions across the powder bed.

## Discussion

Mini-tablets differ fundamentally from conventional tablets due to their small diameter, lower diameter-to-height ratio, and higher surface area-to-volume ratio, which place most particles within a few particle diameters of a confining space [13]. Consequently, compaction occurs in a strongly confinement-dominated environment in which wall proximity and geometric constraints influence nearly the entire powder bed. While earlier studies compared mini- and conventional-tablet behavior using finite element method (FEM) or macroscopic compression analyses, these methods cannot resolve particle-level forces. For example, studies with FEM reported localized low-density regions and shear bands when tablet size changes, suggesting the potential occurrence of tablet defects, but did not resolve contact-level force chains or cohesion gradients [50]. Herein, DEM modeling and analysis was used to quantify normal, tangential, and cohesive interactions within mini-tablets to reveal how aspect ratio and boundary proximity govern micro-mechanics in ways that bulk signatures (e.g., force–displacement curve, yield pressure) alone cannot capture.

### Particle Size Governs the Force Transmission Architecture

Among the variables investigated, particle size exerted the strongest influence on both force magnitude and spatial organization of interparticle forces. Larger particles produced higher peak forces, broader force distributions of normal and tangential forces, and far greater spatial heterogeneity. Because larger particles provided fewer contact points per unit volume, stresses were concentrated along sparse, high-load contacts rather than distributed evenly throughout the bed [51]. Consequently, force transmission became dominated by force chains, where a limited number of particle contacts carried most of the load. Smaller particles, by contrast, with more contacts per unit volume, generated denser contact networks that allowed stress to spread across many interacting particles. As a result, force transmission was more evenly distributed, without the emergence of dominant high-load pathways. These observations showed that particle size directly controls contact stiffness and the development of force chains, whereas weight primarily scaled with the applied load without fundamentally altering the contact network topology.

The particle-level visualizations supported this interpretation, showing that as particle size decreased, force heterogeneity diminished and the compact exhibited more evenly distributed contact forces. This transition from chain-dominated to distributed networks reflected the central role of contact density in determining load transmission under confined compaction [52, 53]. In mini-tablets, where geometric confinement amplifies local stress concentrations, such differences in network structure have pronounced implications for powder densification efficiency and tablet mechanical integrity. Therefore, the improved stress homogenization associated with smaller particles must be balanced against potential reductions in flowability during die filling, emphasizing the importance of formulation optimization.

### Mini-Tablet Geometry Amplifies Boundary Effects

The narrow die cavities characteristic of mini-tablet tooling created an environment in which boundary effects influence virtually the entire powder bed, even though the resulting stress and cohesion fields remained highly heterogeneous in both the axial and radial directions. Spatial analysis of the DEM outcomes showed that normal and tangential forces were consistently higher and more variable near the die wall compared to the interior, reflecting the combined effects of lateral confinement and wall friction. Although clear gradients were present from the center toward the wall, the small diameter of mini-tablets meant that even particles located in the “interior” of the tablet remain only a few particle diameters away from a confining surface. As a result, boundary-driven stresses extended across the whole tablet cross-section rather than being confined to a peripheral region, as typically observed in conventional tablets.

In contrast, cohesive forces were lowest near the boundaries and highest within the compact interior, creating a stress–cohesion mismatch in which the most mechanically stressed regions have the weakest cohesive bonding. This mismatch identified potential structural weak points susceptible to defects such as capping or edge friability, particularly during downstream processes including coating and packaging. The results therefore demonstrated that confinement not only intensified stresses but also redistributed cohesive interactions in a manner unique to narrow die geometries.

### Particle Size Distribution Shapes Force Transmission Networks

The influence of particle size distribution on compaction behavior was investigated at two distinct but interrelated levels: differences in overall particle size range and differences in distribution breadth within a fixed size window. Although both sets of data affected macroscopic compaction behavior, they influenced different aspects of force transmission. For a given particle diameter, normal and tangential forces followed nearly identical force–size trends across the three particle size distributions, with only modest shifts in peak values (Fig. 13). Yet the bulk compact curves differed significantly (Fig. 12A). Therefore, maximum compression force alone was insufficient to interpret particle size distribution effects because peak forces were primarily governed by the largest particles present, whereas bulk behavior depended on how forces were organized across the network. Importantly, these two particle size distribution characteristics operated through different mechanisms: overall size range determined the maximum force potential through the presence of large particles, whereas distribution breadth controlled how those forces were redistributed across the contact network.

Variation in particle size range primarily altered the magnitude and localization of force transmission. In the widest range (90–710 µm), force transmission was highly localized, with the load concentrated at a small subset of the largest particles. These contacts formed rigid load-bearing pathways that limited the ability of the powder bed to reorganize during compression. As the overall size range narrowed to 150–425 µm and then to 212–300 µm, force magnitudes decreased and stress transmission became progressively more distributed among a greater fraction of particles. These changes also led to differences in RE/PE ratios, indicating that in addition to shifts in force magnitude, rearrangement dynamics during compaction was also altered, with a greater proportion of the compaction work being attributed to rearrangement-related processes.

In contrast, variation in distribution breadth within a fixed size window had minimal influence on local force scaling but substantially altered the organization of the force network. Narrow distributions formed mechanically rigid, chain-like skeletons spanning the cross-section. These chains limited rearrangement, maintained load paths even at modest strains, and promoted early stiffening of the bed, so that further densification required large additional punch forces. In contrast, for broader distributions, particles of different sizes participated in the network so that the same force was distributed across a larger number of contacts, producing a more diffuse, heterogeneous but less chain-dominated network that could more easily break, reform, and reorganize under load. In this case, the more effective rearrangement and void filling provided by small and intermediate particles reduced the overall resistance of the bed to densification, so the same displacement could be achieved with a smaller applied punch force, even though the largest particles still carried comparable peak contact forces [54]. The slightly elevated RE/PE ratio in broader systems therefore reflects prolonged network rearrangement rather than increased resistance to compaction, consistent with the more distributed and adaptable force networks observed at the particle level.

### Implications for Mini-Tablet Preparation

Collectively, these findings demonstrated that particle size distribution range governed the maximum force potential through the presence of large particles, whereas distribution breadth governed the adaptability and topology of the force network. Because peak forces were primarily dictated by particle size, evaluation of particle size distribution effects based solely on maximum interparticle force can obscure critical differences in network organization and rearrangement dynamics. Under confined mini-tablet conditions, where boundary effects amplified stress heterogeneity, these network-level differences played a dominant role in determining densification efficiency and mechanical attributes.

From a formulation perspective, achieving robust mini-tablet compaction requires balancing competing factors [31, 35, 36]. Smaller particles improve stress homogeneity but may impair flowability, whereas larger particles enhance flow but increase force localization. Similarly, broader particle size distributions facilitate rearrangement and reduce compression force but may introduce localized high-force contacts associated with large particles. DEM analysis provides a framework to evaluate these trade-offs by linking particle-scale mechanics to macroscopic compaction performance.

Importantly, these findings help explain why similar or even slightly higher compression forces do not necessarily translate into higher tensile strength in mini-tablets. Although the 5 mm fill depth exhibited a slightly higher average peak compression force, the resulting mini-tablets showed lower in-die and out-of-die solid fractions and reduced tensile strength. This behavior reflects less efficient densification arising from increased stress heterogeneity, force-chain localization, and axial density gradients under confined conditions, rather than from increased elastic recovery alone. Consequently, final mechanical strength is governed by the organization and redistribution of interparticle forces within the compact, rather than by peak force or force–displacement behavior alone. These results highlight the importance of particle-scale force-network characteristics in determining the mechanical integrity of mini-tablets under highly confined geometries.

## Conclusion

This study used DEM simulations to investigate how powder fill weight, particle size, and particle size distribution influenced force transmission during compaction in narrow die cavities. While increasing fill weight led to higher compaction forces and larger average interparticle forces, the results showed that particle size played a much more dominant role in governing both the magnitude and heterogeneity of force transmission. Larger particles consistently carried higher normal, tangential, and cohesive forces, indicating that load was concentrated in a limited number of contacts rather than being evenly shared across the powder bed.

Spatial analyses revealed strong axial and radial heterogeneity in force transmission, with normal and tangential forces intensifying near the die wall due to lateral confinement and friction, while cohesive forces were weakest at the boundaries and strongest in the compact interior. These boundary-induced gradients highlighted the importance of confinement effects in narrow dies and helped explain the development of mechanically vulnerable regions that may contribute to defects in compacted mini-tablets.

For polydisperse systems, the results demonstrated that particle size distribution width had a limited effect on the maximum forces experienced by individual particles, which were primarily governed by the largest particles in the system. However, distribution breadth had a profound impact on the macroscopic compaction response. Narrow size distributions promoted the formation of localized force chains and rigid load-bearing skeletons that resisted densification, leading to higher compaction forces. In contrast, wider distributions facilitated particle rearrangement and more distributed force networks, allowing the applied load to be shared across a larger number of contacts and resulting in significantly lower bulk compaction forces.

Overall, this work shows that macroscopic compaction behavior should not be inferred solely from particle-scale force magnitudes but instead depends critically on how those forces are organized spatially within the powder bed. By explicitly linking particle size, size distribution, and force network structure to bulk mechanical response, this study provides mechanistic insights into powder compaction under confined conditions and offers guidance for the rational design of mini-tablets.

## Supplementary Information

Below is the link to the electronic supplementary material.

ESM1 (DOCX 3.80 MB)

## Data Availability

The data supporting the findings of this study are available from the corresponding author upon request.
