# Supplementary material for: Force Distribution and Contact Mechanics in Mini-Tablet Compaction: A Discrete Element Method Study
Source: Pharm Res. 2026 Jun 18;43(7):2361–79. doi: 10.1007/s11095-026-04127-y (PMC13545058; doi:10.1007/s11095-026-04127-y)
Supplement: Supplementary file 1 [file 11095_2026_4127_MOESM1_ESM.docx]

# Supporting information for the manuscript entitled

**Force Distribution and Contact Mechanics in Mini‑Tablet Compaction: A Discrete Element Method Study**

Saeed Najafian^1,2^, Trong Bien Tran^3^, Bodhisattwa Chaudhuri^1,2,4^, Tze Ning Hiew^3*^

1. Department of Pharmaceutical Sciences, School of Pharmacy and Pharmaceutical Sciences, University of Connecticut, Storrs, CT 06269, USA
2. Department of Chemical and Biomolecular Engineering, College of Engineering, University of Connecticut, Storrs, CT 06269, USA
3. Department of Pharmaceutical Sciences and Experimental Therapeutics, College of Pharmacy, The University of Iowa, Iowa City, IA 52242, USA
4. Institute of Material Sciences, University of Connecticut, Storrs, CT 06269, USA

## Particle size distributions for truncated ranges

**Table S1.** **Narrow particle size distribution (212–300 µm; σ = 0.05)**

| **Particle size (μm)** | **Cumulative mass fraction (%)** |
| --- | --- |
| 212 | 0.000 |
| 224 | 0.862 |
| 236 | 9.204 |
| 250 | 43.071 |
| 260 | 72.917 |
| 270 | 91.405 |
| 280 | 98.203 |
| 300 | 100.000 |

**Table S2.** **Intermediate particle size distribution (150–425 µm; σ = 0.1)**

| **Particle size (μm)** | **Cumulative mass fraction (%)** |
| --- | --- |
| 150 | 0 |
| 160 | 0.000 |
| 180 | 0.000 |
| 200 | 0.036 |
| 212 | 0.989 |
| 224 | 4.025 |
| 250 | 11.562 |
| 280 | 46.056 |
| 300 | 84.950 |
| 315 | 95.766 |
| 355 | 98.652 |
| 400 | 99.967 |
| 425 | 100.000 |

**Table S3. Wide and W_narrow_ particle size distribution (90–710 µm; σ = 0.25)**

| **Particle size (μm)** | **Cumulative mass fraction (%)** |
| --- | --- |
| 90 | 0.000 |
| 100 | 0.009 |
| 106 | 0.024 |
| 112 | 0.055 |
| 125 | 0.241 |
| 140 | 0.903 |
| 150 | 1.840 |
| 160 | 3.365 |
| 180 | 8.717 |
| 200 | 17.440 |
| 212 | 24.077 |
| 224 | 31.434 |
| 250 | 48.233 |
| 280 | 65.874 |
| 300 | 75.333 |
| 315 | 81.062 |
| 355 | 91.283 |
| 400 | 96.682 |
| 425 | 98.117 |
| 450 | 98.949 |
| 500 | 99.683 |
| 560 | 99.929 |
| 600 | 99.975 |
| 630 | 99.989 |
| 710 | 100.000 |

**Table S4. W_intermediate_ particle size distribution (90–710 µm; σ = 0.4)**

| **Particle size (μm)** | **Cumulative mass fraction (%)** |
| --- | --- |
| 90 | 0.000 |
| 100 | 0.535 |
| 106 | 1.008 |
| 112 | 1.617 |
| 125 | 3.458 |
| 140 | 6.554 |
| 150 | 9.198 |
| 160 | 12.273 |
| 180 | 19.496 |
| 200 | 27.690 |
| 212 | 32.833 |
| 224 | 38.006 |
| 250 | 48.884 |
| 280 | 60.188 |
| 300 | 66.736 |
| 315 | 71.094 |
| 355 | 80.504 |
| 400 | 87.809 |
| 425 | 90.701 |
| 450 | 92.954 |
| 500 | 96.044 |
| 560 | 98.135 |
| 600 | 98.946 |
| 630 | 99.364 |
| 710 | 100.000 |

**Table S5. W_wide_ particle size distribution (90–710 µm; σ = 1)**

| **Particle size (μm)** | **Cumulative mass fraction (%)** |
| --- | --- |
| 90 | 0.000 |
| 100 | 3.724 |
| 106 | 5.948 |
| 112 | 8.155 |
| 125 | 12.858 |
| 140 | 18.106 |
| 150 | 21.482 |
| 160 | 24.753 |
| 180 | 30.965 |
| 200 | 36.740 |
| 212 | 39.999 |
| 224 | 43.110 |
| 250 | 49.367 |
| 280 | 55.832 |
| 300 | 59.736 |
| 315 | 62.471 |
| 355 | 69.035 |
| 400 | 75.328 |
| 425 | 78.401 |
| 450 | 81.210 |
| 500 | 86.147 |
| 560 | 91.074 |
| 600 | 93.868 |
| 630 | 95.746 |
| 710 | 100.000 |

## Tangential force distribution with respect to powder fill weight

Figure S1. Axial tangential force distributions for (A) 7.5 mg, (B) 8.0 mg, (C) 8.5 mg, and (D) 9.0 mg samples showed a systematic increase in median force with increasing weight. Radial tangential force distributions for (E) 7.5 mg, (F) 8.0 mg, (G) 8.5 mg, and (H) 9.0 mg exhibited a general increase in force magnitude with radial distance from the cylinder axis.

Figure S2. Particle-level tangential force distributions within a compact cross section at (A) 7.5 mg, (B) 8.0 mg, (C) 8.5 mg, and (D) 9.0 mg. Tangential forces exhibited localized high-force regions near boundaries, reflecting frictional interactions and lateral confinement effects. These regions became more widespread and intense as tablet weight increases.

## Tangential force distribution with respect to particle size in monodisperse systems

**Figure S3. Axial tangential force distributions for (A) 100 µm, (B) 150 µm, (C) 200 µm, and (D) 250 µm samples showed a systematic increase in median force with increasing particle size. Radial tangential force distributions for (E) 100 µm, (F) 150 µm, (G) 200 µm, and (H) 250 µm also showed increasing mean with increase in particle size.**

**Figure S4. Particle-level tangential force distributions within a compact cross section at (A) 100 µm, (B) 150 µm, (C) 200 µm, and (D) 250 µm.** Tangential force distributions across particle sizes showed that larger particles exhibited slightly higher variability and localized regions of elevated tangential force, whereas smaller particles displayed more uniform tangential force fields. This trend indicates reduced interparticle sliding and more stable contact configurations at smaller particle sizes.

## Tangential force distribution with respect to particle size in polydisperse systems

**Figure S5. Particle-level tangential force distributions within a compact cross section for 90-710µm, 150–425 µm, and 212–300 µm particle size distributions.** Tangential force distributions illustrated a strong dependence on particle size spread. In the widest size range (90–710 µm), tangential forces were highly localized and concentrated at contacts involving the largest particles, forming distinct load-bearing pathways. As the size distribution narrowed, the tangential force network became more uniformly distributed across the powder bed, with reduced localization and more particles contributing to frictional load transfer.

**Figure S6. Particle-level tangential force distributions within a compact cross section for W_narrow_, W_intermediate_, and W_wide_ within the 90–710 µm size window.** In the narrow distribution, tangential forces were highly localized at a single dominant large-particle contact, while surrounding smaller particles experienced minimal frictional loading. As the distribution breadth increased, tangential forces became progressively more distributed across the powder bed, with multiple medium-to-large particles contributing to frictional load transfer.

## Appendix A

### Sensitivity Analysis

A sensitivity analysis was performed to evaluate the influence of key model parameters on the compaction response. Three parameters were selected for this purpose: Young’s modulus, surface energy, and plasticity ratio. A base case (Case A) was first established using parameter values that were previously calibrated and experimentally validated, providing the best agreement with measured data. Subsequently, each parameter was varied individually while keeping the others constant, allowing for a systematic assessment of their impact (Table A1). The selected ranges were chosen to represent reasonable variations around the calibrated baseline without introducing nonphysical behavior.

The axial normal force distribution was then compared across all cases. The results show that both the magnitude and spatial distribution of axial forces remain nearly identical to the base case for all parameter variations considered (Figure A1). In addition, the overall trends are preserved, with no significant deviation observed in the force profiles. These findings suggest that, within the investigated parameter ranges, the model predictions are relatively insensitive to moderate changes in Young’s modulus, surface energy, and plasticity ratio. This indicates that the compaction behavior captured in the simulations is robust and not strongly dependent on small uncertainties in these input parameters.

Table A1. DEM model parameter sets used in the sensitivity analysis.

| **Case** | **Young Modulus (N/m^2^)** | **Surface Energy (J/m^2^)** | **Plasticity Ratio** |
| --- | --- | --- | --- |
| A (Base Case) | 4.4 × 10^9^ | 1 | 0.99 |
| B | 4.4 × 10^9^ | 2 | 0.99 |
| C | 4.4 × 10^9^ | 0.5 | 0.99 |
| D | 4.4 × 10^9^ | 1 | 0.9 |
| E | 4.4 × 10^9^ | 1 | 0.8 |
| F | 4.8 × 10^9^ | 1 | 0.99 |
| G | 4.0 × 10^9^ | 1 | 0.99 |

**Figure A1.** **Sensitivity analysis of material parameters used in the DEM simulations.** The base case (Case A) corresponds to the validated parameter set with the best agreement to measurements. Cases B–G represent systematic variations in surface energy, plasticity ratio, and Young’s modulus relative to the base case, with one parameter varied at a time. The results show that both the magnitude and spatial distribution of axial forces remain nearly identical to the base case for all parameter variations considered.
